# Supplementary material for: Demographic characteristics, long-term health conditions and healthcare experiences of 6333 trans and non-binary adults in England: nationally representative evidence from the 2021 GP Patient Survey
Source: BMJ Open. 2023 Feb 2;13(2):e068099. doi: 10.1136/bmjopen-2022-068099 (PMC9895920; doi:10.1136/bmjopen-2022-068099)

BMJ Publishing Group Limited (BMJ) disclaims all liability and responsibility arising from any reliance placed on this supplemental material which has been supplied by the author(s)

Appendix Table 1. Patient experience, question wording and categorisation for analysis. Reproduced directly from Saunders et al Sexes 2022, 3(3), 325-335; https://doi.org/10.3390/sexes3030025

**Overall experience**	
Overall experience	*Overall, how would you describe your experience of your GP practice?* Very good, Fairly good, compared with Neither good nor poor, Fairly poor, and Very poor
Overall experience of making an appointment	*Overall, how would you describe your experience of making an appointment?* Very good, Fairly good, compared with Neither good nor poor, Fairly poor, and Very poor
**Before trying to make an appointment**	
Tried self-management	*Before you tried to get this appointment, did you do any of the following?* Spoke to a pharmacist, Tried to treat myself/the person I was making this appointment for (for example with medication), compared with all other question respondents *
Asked friends or family	*Before you tried to get this appointment, did you do any of the following?* Asked for advice from a friend or family member, compared with all other question respondents *
Tried online, telephone or other NHS services	*Before you tried to get this appointment, did you do any of the following?* Used an online NHS service (including NHS 111 online), Called an NHS helpline, such as NHS 111, Contacted or used another NHS service, compared with all other question respondents *
Tried online or other non-NHS services	*Before you tried to get this appointment, did you do any of the following?* Used a non-NHS online service, or looked online for information, Tried to get information or advice elsewhere (from a non-NHS service), compared with all other question respondents *
**Access**	
Found GP practice website easy to use	*How easy is it to use your GP practice's website to look for information or access services?* Very easy, Fairly easy, compared with Not very easy, and not at all easy. **
Tried to make an appointment in the last 6 months	*When did you last try to make a general practice appointment, either for yourself or for someone else?* In the past 3 months, Between 3 and 6 months ago, compared with Between 6 and 12 months ago, More than 12 months ago, and I haven't tried to make an appointment since being registered with my current GP practice.
Getting through on the phone	*Generally, how easy is it to get through to someone at your GP practice on the phone?* Very easy, Fairly easy, compared with Not very easy, Not at all easy
Helpful receptionists	*How helpful do you find the receptionists at your GP practice?* Very helpful, Fairly helpful, compared with Not very helpful, and Not at all helpful
Offered a choice when booking appointment	*On this occasion, were you offered any of the following choices of appointment?* Yes, a choice of place (for an appointment in person), Yes, a choice of time or day, Yes, a choice of healthcare professional, Yes a choice of type of appointment (phone call, online, video call, in person), compared with None of these
Satisfied with appointment times available	*How satisfied are you with the general practice appointment times that are available to you?* Very satisfied, Fairly satisfied, compared with Neither satisfied nor dissatisfied, Fairly dissatisfied, and Very dissatisfied
Offered an acceptable appointment	*Were you satisfied with the appointment (or appointments) you were offered?* Yes and I accepted an appointment, No, but I still took an appointment, compared with No, and I did not take an appointment, and I was not offered an appointment
Satisfied with appointment offered	*Were you satisfied with the appointment (or appointments) you were offered?* Yes and I accepted an appointment, compared with No, but I still took an appointment, No, and I did not take an appointment, and I was not offered an appointment
Remote appointment (telephone or online)	*What type of appointment did you get?* I got an appointment… "…to speak to someone on the phone", "…to speak to someone online (for example on a video call)" compared with "…to see someone at my GP practice", "…to see someone at another general practice location", "…for a home visit"
**Continuity**	
Have a preferred GP	*Is there a particular GP you usually prefer to see or speak to?* Yes, for all appointments, Yes for some appointments but not others, compared with No ***
Able to see preferred GP	*How often do you see or speak to your preferred GP when you would like to?* Always or almost always, A lot of the time, compared with Some of the time, and Never or almost never. ****
**Communication**	
Involved in decisions about care and treatment	*During your last general practice appointment, were you involved as much as you wanted to be in decisions about your care and treatment?* Yes, definitely, Yes, to some extent, compared with No, not at all
Had MH needs in last appointment	*During your last general practice appointment, did you feel that the healthcare professional recognised and/or understood any mental health needs that you might have had?* Yes, definitely, Yes to some extent, No, not at all, compared with I did not have any mental health needs
MH needs recognised and understood	*During your last general practice appointment, did you feel that the healthcare professional recognised and/or understood any mental health needs that you might have had?* Yes, definitely, Yes to some extent, compared with No, not at all
Confidence and trust	*During your last general practice appointment, did you have confidence and trust in the healthcare professional you saw or spoke to?* Yes, definitely, Yes, to some extent, compared with No, not at all
Needs were met	*Thinking about the reason for your last general practice appointment, were your needs met?* Yes, definitely, Yes, to some extent, compared with No, not at all

* Only including respondents who have tried to make an appointment with their GP. ** Only including respondents who have tried to use their GP practice's website. *** Excluding responses from people with only one GP in their practice. **** Only including people with a preference for a particular GP.

Appendix Table 2. Respondent characteristics; row percentages (all responses – comparison with Table 1 in the main paper which presents the same data with column percentages)

	All trans and non-binary respondents (n,%)	All other survey respondents (n,%)
**All respondents (n=840,691)**	6333 (0.9)	834358 (99.1)
**Gender (n=835,561)**		
Female	1708 (0.4)	468958 (99.6)
Male	1971 (0.5)	359266 (99.5)
Non-binary	1220 (100)	
Prefer to self-describe	1047 (100)	
Prefer not to say	103 (7.4)	1288 (92.6)
**Trans status (n=834,746)**		
Gender identity the same as sex registered at birth	957 (0.1)	825209 (99.9)
Gender identity different from sex registered at birth	4642 (100)	
Prefer not to say	644 (16.4)	3294 (83.6)
**Age (n=833,526)**		
16-24	628 (1.5)	41162 (98.5)
25-34	838 (1.2)	69764 (98.8)
35-44	1109 (1.1)	100108 (98.9)
45-54	1155 (0.8)	137231 (99.2)
55-64	1080 (0.6)	174512 (99.4)
65-74	827 (0.5)	172155 (99.5)
75-84	573 (0.4)	132384 (99.6)
**Ethnicity (n=834,261)**		
White	3343 (0.5)	702888 (99.5)
Asian	176 (1.6)	10780 (98.4)
Black	1467 (2.0)	70305 (98.0)
Mixed	482 (1.7)	27612 (98.3)
Other	727 (4.2)	16481 (95.8)
**Sexual orientation (n=820,113)**		
Heterosexual	2682 (0.4)	754746 (99.6)
Lesbian / gay	310 (2.6)	11452 (97.4)
Bisexual	462 (5.3)	8186 (94.7)
Other	752 (10.8)	6235 (89.2)
Prefer not to say	1614 (4.6)	33674 (95.4)
**Deprivation (n=840,691)**		
Most deprived	2211 (1.3)	162804 (98.7)
2	1650 (1.0)	167119 (99.0)
3	1117 (0.6)	172925 (99.4)
4	791 (0.5)	169938 (99.5)
Least deprived	561 (0.3)	161387 (99.7)

Appendix Table 3. Respondent characteristics; (for comparison with Table 1 from main paper; this version includes only the 827,696 responses from the multivariable analysis sample with complete data from all covariates included in multivariable analysis with 6,091 trans and non-binary respondents)

	All trans and non-binary respondents (n, %)	All other survey respondents (n, %)
**All respondents**	6091	827696
**Gender**		
Female	1659 (28.5)	461698 (56.5)
Male	1903 (32.6)	354519 (43.4)
Non-binary	1189 (20.4)	
Prefer to self-describe	981 (16.8)	
Prefer not to say	99 (1.7)	1239 (0.2)
**Trans status**		
Gender identity the same as sex registered at birth	920 (15.3)	813715 (99.6)
Gender identity different from sex registered at birth	4493 (74.7)	
Prefer not to say	599 (10.0)	3073 (0.4)
**Age**		
16-24	622 (10.2)	40967 (5.0)
25-34	826 (13.6)	69415 (8.4)
35-44	1090 (17.9)	99493 (12.1)
45-54	1127 (18.5)	136323 (16.6)
55-64	1061 (17.4)	173233 (21.1)
65-74	809 (13.3)	170965 (20.8)
75-84	556 (9.1)	131209 (16.0)
**Ethnicity**		
White	3300 (54.2)	698007 (85.0)
Asian	170 (2.8)	10656 (1.3)
Black	1442 (23.7)	69465 (8.5)
Mixed	474 (7.8)	27260 (3.3)
Other	705 (11.6)	16217 (2.0)
**Deprivation**		
Most deprived	2127 (34.9)	159640 (19.4)
2	1591 (26.1)	164302 (20.0)
3	1072 (17.6)	170406 (20.7)
4	760 (12.5)	167823 (20.4)
Least deprived	541 (8.9)	159434 (19.4)

Appendix table 4. All age stratified OR and 95%CI (from the model underpinning Figure 1)

	16-24	25-34	35-44	45-54	55-64	65-74	75+
Autism or autism spectrum condition	4.4 (3.4 - 5.6)	5.5 (4.1 - 7.3)	5.4 (3.7 - 7.8)	7.1 (4.9 - 10.3)	10.5 (7.0 - 15.7)	10.5 (5.6 - 20.0)	8.0 (3.3 - 19.6)
Alzheimer's disease or other cause of dementia	2.7 (0.4 - 20.2)	15.2 (8.1 - 28.6)	12.7 (7.5 - 21.4)	6.6 (3.6 - 11.8)	4.7 (2.7 - 8.1)	2.9 (1.7 - 4.8)	1.3 (0.8 - 1.9)
A learning disability	1.9 (1.4 - 2.7)	2.2 (1.6 - 3.0)	2.5 (1.7 - 3.6)	3.7 (2.6 - 5.1)	4.0 (2.8 - 5.8)	7.1 (4.5 - 11.4)	4.6 (2.0 - 10.3)
A mental health condition	4.4 (3.7 - 5.3)	3.0 (2.6 - 3.6)	1.2 (1.0 - 1.5)	1.4 (1.2 - 1.7)	1.5 (1.2 - 1.8)	1.8 (1.4 - 2.4)	1.8 (1.1 - 2.9)
A stroke (which affects your day to day life)	1.6 (1.0 - 2.4)	3.4 (1.1 - 10.7)	3.2 (1.5 - 6.8)	2.2 (1.3 - 3.8)	1.2 (0.7 - 2.1)	2.0 (1.3 - 3.1)	1.6 (1.0 - 2.4)
Blindness or partial sight	2.8 (1.5 - 5.3)	2.5 (1.3 - 4.8)	1.8 (1.0 - 3.4)	2.2 (1.4 - 3.5)	1.6 (1.0 - 2.5)	1.7 (1.1 - 2.6)	1.1 (0.7 - 1.6)
Kidney or liver disease	2.0 (0.7 - 5.4)	0.7 (0.3 - 1.9)	2.0 (1.3 - 3.0)	1.4 (1.0 - 2.1)	1.3 (0.9 - 1.9)	1.1 (0.7 - 1.6)	1.5 (1.0 - 2.1)
A neurological condition such as epilepsy	1.3 (0.6 - 2.5)	2.1 (1.4 - 3.2)	0.9 (0.5 - 1.5)	1.5 (1.0 - 2.2)	1.1 (0.7 - 1.7)	1.5 (0.9 - 2.4)	0.9 (0.4 - 2.0)
Deafness or hearing loss	1.1 (0.5 - 2.5)	2.5 (1.5 - 4.0)	1.9 (1.3 - 2.9)	1.4 (1.0 - 2.0)	1.4 (1.0 - 1.8)	1.1 (0.9 - 1.5)	0.9 (0.7 - 1.1)
Diabetes	0.2 (0.0 - 1.7)	1.2 (0.8 - 2.0)	1.5 (1.1 - 1.9)	1.4 (1.1 - 1.7)	1.1 (0.9 - 1.3)	1.3 (1.1 - 1.6)	1.2 (0.9 - 1.4)
Another long-term condition or disability	1.8 (1.4 - 2.3)	1.7 (1.3 - 2.1)	1.0 (0.8 - 1.2)	0.8 (0.6 - 0.9)	1.1 (0.9 - 1.3)	1.1 (0.9 - 1.4)	1.3 (1.0 - 1.7)
A heart condition such as angina or atrial fibrillation	1.6 (0.6 - 3.8)	1.3 (0.6 - 2.8)	1.2 (0.7 - 2.2)	1.0 (0.6 - 1.5)	1.1 (0.9 - 1.4)	1.0 (0.8 - 1.3)	0.9 (0.7 - 1.2)
A breathing condition such as asthma or COPD	1.2 (0.9 - 1.6)	1.0 (0.8 - 1.3)	0.8 (0.6 - 1.1)	1.0 (0.8 - 1.2)	0.9 (0.7 - 1.1)	1.0 (0.8 - 1.2)	1.0 (0.7 - 1.3)
Arthritis or ongoing problem with back or joints	2.5 (1.7 - 3.7)	1.4 (1.1 - 2.0)	1.3 (1.0 - 1.6)	0.8 (0.7 - 1.0)	0.8 (0.7 - 1.0)	0.8 (0.7 - 1.0)	1.0 (0.8 - 1.2)
High blood pressure	1.3 (0.4 - 4.0)	1.4 (0.8 - 2.3)	1.3 (1.0 - 1.7)	1.0 (0.8 - 1.2)	0.9 (0.7 - 1.0)	0.9 (0.8 - 1.0)	0.8 (0.7 - 1.0)
Cancer (diagnosis or treatment in the last 5 years)	2.4 (0.6 - 9.8)	1.1 (0.4 - 3.5)	1.3 (0.7 - 2.5)	0.7 (0.4 - 1.2)	1.1 (0.8 - 1.5)	0.7 (0.5 - 1.0)	0.8 (0.5 - 1.1)
I do not have any long term conditions	0.3 (0.2 - 0.3)	0.5 (0.4 - 0.6)	0.8 (0.7 - 1.0)	0.9 (0.8 - 1.0)	0.9 (0.8 - 1.0)	0.9 (0.8 - 1.1)	1.2 (0.9 - 1.6)

Appendix figure 1. Dementia, learning disability, blindness and neurological conditions. Long-term condition adjusted prevalence in trans and non-binary adults follows the same pattern as all other survey respondents, but prevalence shifted upwards with higher prevalence at each age

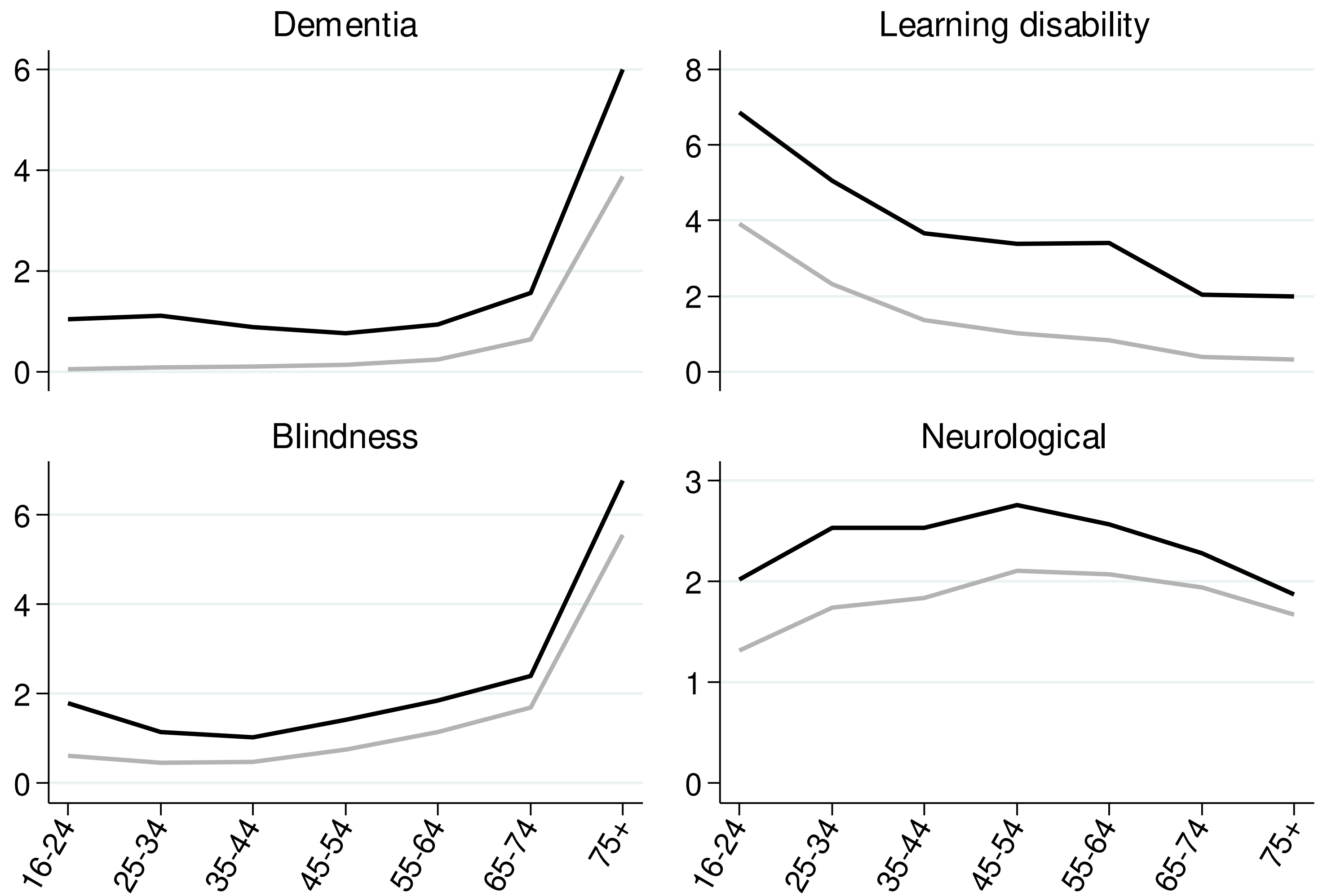

Appendix figure 2. Stroke, kidney or liver problems and diabetes; disparities increase with age, with trans and non-binary adults having increasingly higher prevalence compared with all other survey respondents at older age

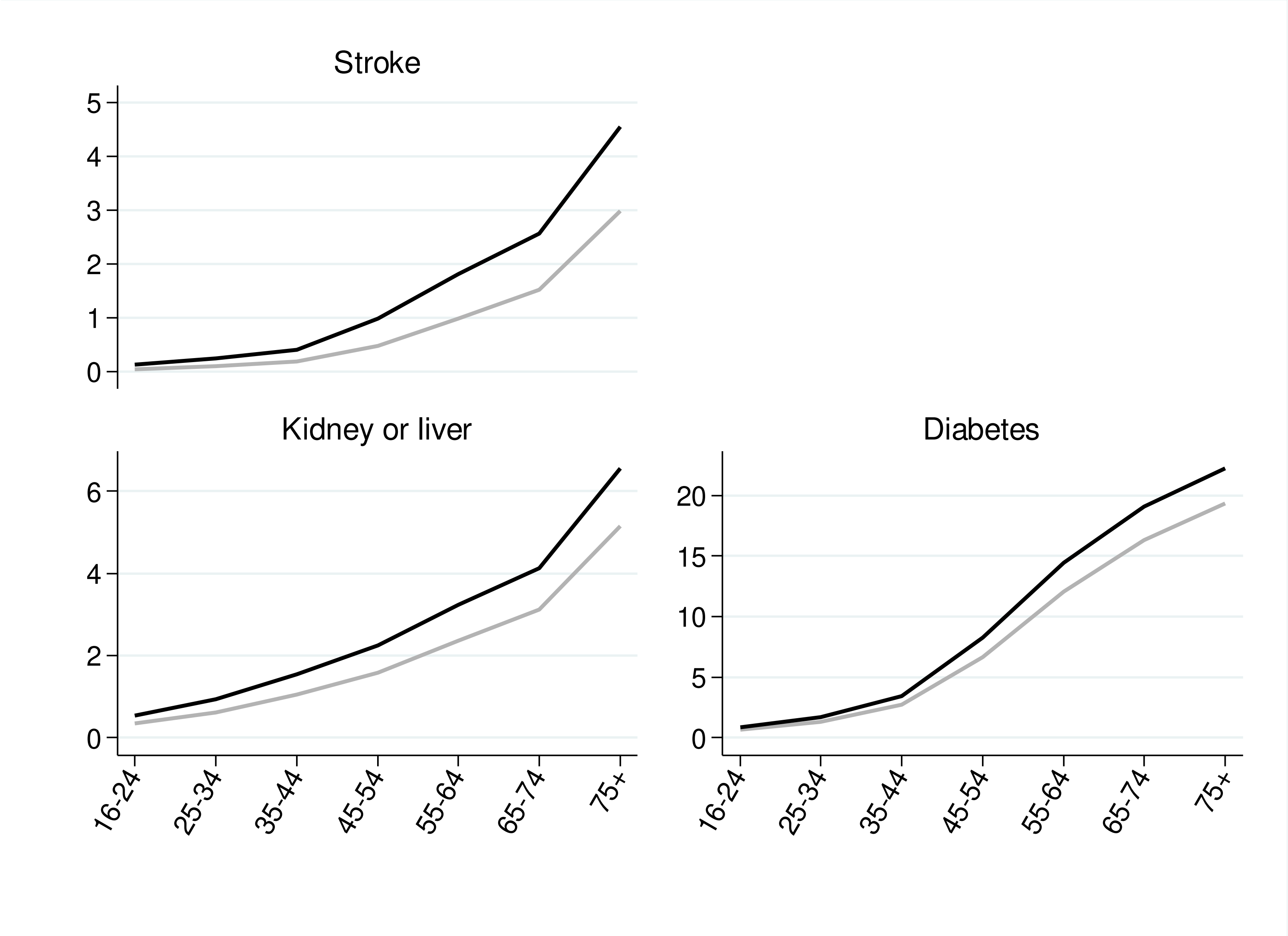

Appendix figure 3. Deafness, heart conditions, breathing problems, high blood pressure and cancer; adjusted prevalence in trans and non-binary adults at older ages is lower than prevalence for all other survey respondents

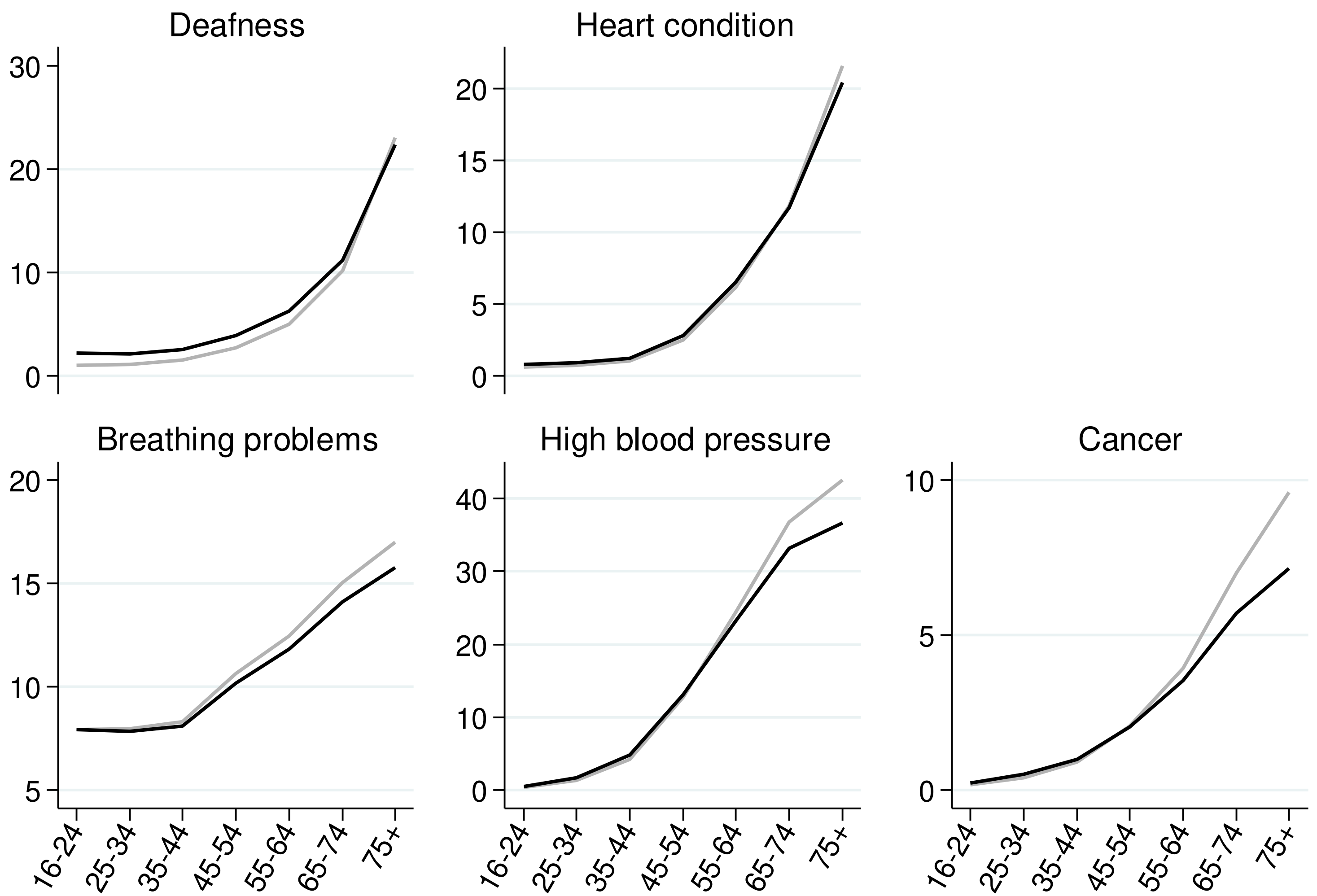

Supplement: Supplementary data [file bmjopen-2022-068099supp001.pdf]
