# Supplementary material for: Demographic characteristics, long-term health conditions and healthcare experiences of 6333 trans and non-binary adults in England: nationally representative evidence from the 2021 GP Patient Survey
Source: BMJ Open. 2023 Feb 2;13(2):e068099. doi: 10.1136/bmjopen-2022-068099 (PMC9895920; doi:10.1136/bmjopen-2022-068099)
Supplement: Author's manuscript [file bmjopen-2022-068099.draft_revisions.pdf]

BMJ Open is committed to open peer review. As part of this commitment we make the peer review history of every article we publish publicly available.

When an article is published we post the peer reviewers' comments and the authors' responses online. We also post the versions of the paper that were used during peer review. These are the versions that the peer review comments apply to.

The versions of the paper that follow are the versions that were submitted during the peer review process. They are not the versions of record or the final published versions. They should not be cited or distributed as the published version of this manuscript.

BMJ Open is an open access journal and the full, final, typeset and author-corrected version of record of the manuscript is available on our site with no access controls, subscription charges or pay-per-view fees (http://bmjopen.bmj.com).

If you have any questions on BMJ Open's open peer review process please email info.bmjopen@bmj.com

BMJ Open

Long-term health conditions and experiences of primary care: Nationally representative evidence from the 2021 GP Patient Survey including responses from 6,333 trans and non-binary adults in England

Journal:	*BMJ Open*
Manuscript ID	bmjopen-2022-068099
Article Type:	Original research
Date Submitted by the Author:	12-Sep-2022
Complete List of Authors:	Saunders, Catherine; University of Cambridge, Department of Public Health and Primary Care<br>Berner, Alison; Tavistock and Portman NHS Foundation Trust,<br>Lund, Jenny; University of Cambridge, Primary Care Unit, Department of Public Health & Primary Care<br>Mason, Amy M; University of Cambridge, Department of Public Health and Primary Care<br>Oakes-Monger, Tash; NHS Improvement<br>Roberts, Meg; University of Cambridge<br>Smith, Jack; Lifestrong<br>Duschinsky, Robbie; University of Cambridge Primary Care Unit
Keywords:	PRIMARY CARE, Quality in health care < HEALTH SERVICES ADMINISTRATION & MANAGEMENT, HEALTH SERVICES ADMINISTRATION & MANAGEMENT

SCHOLARONE™
Manuscripts

BMJ

I, the Submitting Author has the right to grant and does grant on behalf of all authors of the Work (as defined in the below author licence), an exclusive licence and/or a non-exclusive licence for contributions from authors who are: i) UK Crown employees; ii) where BMJ has agreed a CC-BY licence shall apply, and/or iii) in accordance with the terms applicable for US Federal Government officers or employees acting as part of their official duties; on a worldwide, perpetual, irrevocable, royalty-free basis to BMJ Publishing Group Ltd ("BMJ") its licensees and where the relevant Journal is co-owned by BMJ to the co-owners of the Journal, to publish the Work in this journal and any other BMJ products and to exploit all rights, as set out in our licence.

The Submitting Author accepts and understands that any supply made under these terms is made by BMJ to the Submitting Author unless you are acting as an employee on behalf of your employer or a postgraduate student of an affiliated institution which is paying any applicable article publishing charge ("APC") for Open Access articles. Where the Submitting Author wishes to make the Work available on an Open Access basis (and intends to pay the relevant APC), the terms of reuse of such Open Access shall be governed by a Creative Commons licence – details of these licences and which Creative Commons licence will apply to this Work are set out in our licence referred to above.

Other than as permitted in any relevant BMJ Author's Self Archiving Policies, I confirm this Work has not been accepted for publication elsewhere, is not being considered for publication elsewhere and does not duplicate material already published. I confirm all authors consent to publication of this Work and authorise the granting of this licence.

For peer review only

Long-term health conditions and experiences of primary care: Nationally representative evidence from the 2021 GP Patient Survey including responses from 6,333 trans and non-binary adults in England

Catherine L Saunders,[1,2*] Alison Berner,[3,4] Jenny Lund,[1] Amy M Mason,[5] Tash Oakes-Monger,[6] Meg Roberts,[7] Jack Smith,[8] Robbie Duschinsky[1]

1. Primary Care Unit, Department of Public Health and Primary Care, University of Cambridge, Cambridge, UK
2. Cambridge Public Health, University of Cambridge, Cambridge, UK
3. Barts Cancer Institute, Queen Mary University of London, London, UK
4. Gender Identity Clinic, Tavistock and Portman NHS Foundation Trust< London, UK
5. British Heart Foundation Cardiovascular Epidemiology Unit, Department of Public Health and Primary Care, University of Cambridge, Cambridge, UK
6. NHS England and NHS Improvement, London, UK
7. Newnham College, University of Cambridge, UK
8. Lifestrong, Wolverhampton, UK

*Corresponding author; ks659@medschl.cam.ac.uk

For peer review only

Abstract

Objective

In order to address the lack of data on the health and healthcare needs of trans and non-binary adults, NHS England includes questions asking about both gender and trans status in its surveys to support quality improvement programmes.

We used data from the GP Patient Survey to answer the research question: What are the demographic characteristics, health conditions, and healthcare experiences of trans and non-binary adults in England?

Design / Setting

Nationally representative, population-based cross-sectional survey in England.

Method

We calculated weighted descriptive statistics, and using logistic regression explored 15 long-term physical and mental health conditions, and 18 patient experience items, covering overall experience, access, communication and continuity.

Results

Trans and non-binary adults were younger, more likely to be from Asian, Black, Mixed or Other ethnic groups, and more likely to live in more deprived parts of the country. Age-specific patterns of long-term conditions were broadly similar among trans and non-binary adults compared with all other survey respondents, with some variation by condition. Overall, inequalities in long term health conditions were largest for autism, OR (95%CI), 5.8 (5.0 - 6.6), dementia, 3.1 (2.5 - 3.9), learning disabilities, 2.8 (2.4 - 3.2) and mental health, 2.0 (1.9 - 2.2), with variation by age. In healthcare experience, disparities are much greater for interpersonal communication (15 percentage points lower in adjusted analysis), than access. Additionally, trans and non-binary adults report much higher preference for continuity 1.7 (1.6-1.8), with no evidence of any differences in being able to see or speak to a preferred GP.

Conclusion

This research adds up to date evidence about population demographics, health and healthcare needs, to support healthcare improvement for trans and non-binary adults.

Keywords: primary health care, healthcare quality improvement, long-term conditions, patient experience, trans and non-binary

For peer review only

- **What is already known on this topic**

Information about the demographics, health, and healthcare needs of trans and non-binary adults is still emerging; what evidence there is suggests disparities are high

- **What this study adds**

Nationally representative population-based evidence on long-term conditions and healthcare experiences

- **How this study might affect research, practice or policy**

This study provides evidence needed to inform healthcare planning and quality improvement for trans and non-binary adults, particularly in primary care

For peer review only

Introduction

Primary care supports populations across the life course, responding to health and healthcare needs that change over time, and with age, and developments in clinical practice. This includes a workload that spans from caring for an aging population increasingly living with multiple long-term health conditions, to providing healthcare throughout public health crises including the Covid-19 pandemic. Care for people who are trans and non-binary is a further area where GPs are providing health services, while information about the demographics, health, and healthcare needs of the population is still emerging.[1-6]

Addressing gaps in health access and outcomes experienced by minority groups including those with protected characteristics (including gender reassignment) is central to the priorities of the NHS long-term plan.[7,8] Primary care is important for these efforts to prevent ill health and address health inequalities. Clinical guidelines for best practice in trans healthcare to date have only focused on transition or HIV, rather than mental health or primary care.[9] In addition, primary care interventions for trans and non-binary patients have focused on specific areas including ensuring equitable screening access,[10] addressing gaps in secondary care services, and on communication. There is much less evidence about healthcare access and patient experience for non-trans-specific services, and little on the epidemiology of long-term health conditions for trans and non-binary adults, despite these being central dimensions of the work of primary care, and making up the majority of all healthcare contacts.

Data collection within the NHS on trans and non-binary adults and their experiences of healthcare is lacking. To address this evidential need, NHS England is beginning to include questions asking about both gender and trans status in its surveys to support quality improvement programmes.[11,12] We used this data from the GP Patient Survey to answer the research question "what are the demographic characteristics, health conditions, and healthcare experiences of trans and non-binary adults in England?"

For peer review only

Methods

The methods for this secondary data analysis of the GP Patient Survey, including details of the survey data collection,[11] survey tools,[13] measures of gender and trans status, outcomes and outcome coding, statistical analysis and patient and public involvement have been described in full in the pre-registered study protocol. The protocol specifies three research questions; of these, RQ1 ("what are the demographic characteristics, health conditions, and healthcare experiences of trans and non-binary adults in England?") is addressed in this work.[14]

In total 850,206 survey responses were received. We excluded 9515 where neither gender nor trans status were reported (i.e. either "prefer not to say" or missing responses to both questions) and we identified a cohort of trans and non-binary respondents from who selected either non-binary, or a self-definition for their gender, and/or those who affirmed their gender identity was different from their sex registered at birth. There were 6333 trans and non-binary respondents in total. We excluded responses missing age, ethnicity and deprivation for all multivariable analyses, which left 827,696 participants, of whom 6091 were trans and non-binary. We excluded respondents with missing outcome data from the analyses for long-term health conditions and patient experience on an outcome by outcome basis (and report these numbers separately in the results).

In preliminary analyses as per protocol[14] we explored variation by region (the nine government office regions from England), and found little variation across the country except for a slightly higher number of trans and non-binary adults in London, and no impact of region in adjusted models, and so for simplicity we did not include full regional details in our final reporting. We also explored variation across all analyses considering trans and non-binary respondents separately. There are differences in the sizes but not directions of differences between these groups, which warrant further investigation. In this report, in an approach acceptable to the patient and public involvement panel with whom this work was developed,[14] for clarity, we consider all trans and non-binary responses, and additionally responses from people who prefer to self-describe their gender, together in a single group.

To explore demographics of the trans and non-binary population, we described the age, gender, ethnicity, deprivation and sexual orientation of trans and non-binary and all other survey respondents using weighted percentages.

To explore long-term health conditions we described the unadjusted weighted percentage of people living with each of 15 physical or mental health conditions, and in adjusted analysis estimated the odds ratio for reporting each condition for trans and non-binary adults compared with all other survey respondents after adjustment for age, deprivation and ethnicity. In preliminary analyses we additionally considered adjustment for GP practice using a random effect, this had no impact on these models and so we used fixed effect for population-level estimates.

We also explored whether there was evidence for variation in the relationship between being trans and non-binary and each of the 15 long-term health conditions varied by age, ethnicity, deprivation, sexual orientation and region. There was evidence of heterogeneity in the association with trans status for 10 out of the 15 conditions in different age-groups and so we estimated adjusted prevalence

For peer review only

stratified by age using recycled predictions from these models, adjusting to the “average” deprivation and ethnicity of all survey respondents.

To explore the healthcare experience of the trans and non-binary population, we calculated the unadjusted weighted percentage of people reporting a positive experience for 18 patient experience items from 5 domains (overall experience, experience before making an appointment, access, continuity and communication) using the categorisation for each questions’ responses in line with the public reporting of each item.[14] In preliminary analyses we explored both national (fixed) and within practice (random effect) models. Estimates were the same from both approaches and fixed effect estimates are presented here. We also calculated the adjusted percentage of respondents reporting the most positive and the most negative experiences.

All analyses were carried out using Stata 15.3

Patient and Public Involvement

The protocol for this work was developed in collaboration with a trans, non-binary and queer patient and public involvement panel. We met an additional two times online and once in person during this project.

For peer review only

Results

Demographic characteristics

Trans and non-binary adults were younger, more likely to be from Asian, Black, Mixed or Other ethnic groups, less likely to be heterosexual, and are more likely to live in more deprived parts of the country (Table 1, Appendix table 1).

<Insert Table 1 about here>

For peer review only

Table 1. Respondent characteristics (all respondents, n= 840,691)

	All trans and non-binary respondents (n,%)	All other survey respondents (n,%)
**All respondents**	6333 (0.9)	834358 (99.1)
**Gender (n=835561)**		
Female	1708 (0.4)	468958 (99.6)
Male	1971 (0.5)	359266 (99.5)
Non-binary	1220 (100)	
Prefer to self-describe	1047 (100)	
Prefer not to say	103 (7.4)	1288 (92.6)
**Trans status (n=834746)**		
Gender identity the same as sex registered at birth	957 (0.1)	825209 (99.9)
Gender identity different from sex registered at birth	4642 (100)	
Prefer not to say	644 (16.4)	3294 (83.6)
**Age**		
16-24	628 (1.5)	41162 (98.5)
25-34	838 (1.2)	69764 (98.8)
35-44	1109 (1.1)	100108 (98.9)
45-54	1155 (0.8)	137231 (99.2)
55-64	1080 (0.6)	174512 (99.4)
65-74	827 (0.5)	172155 (99.5)
75-84	573 (0.4)	132384 (99.6)
**Ethnicity**		
White	3343 (0.5)	702888 (99.5)
Asian	176 (1.6)	10780 (98.4)
Black	1467 (2.0)	70305 (98.0)
Mixed	482 (1.7)	27612 (98.3)
Other	727 (4.2)	16481 (95.8)
**Sexual orientation**		
Heterosexual	2682 (0.4)	754746 (99.6)
Lesbian / gay	310 (2.6)	11452 (97.4)
Bisexual	462 (5.3)	8186 (94.7)
Other	752 (10.8)	6235 (89.2)
Prefer not to say	1614 (4.6)	33674 (95.4)
**Deprivation**		
Most deprived	2211 (1.3)	162804 (98.7)
2	1650 (1.0)	167119 (99.0)
3	1117 (0.6)	172925 (99.4)
4	791 (0.5)	169938 (99.5)
Least deprived	561 (0.3)	161387 (99.7)

For peer review only

Long-term conditions

The weighted, unadjusted, percentages of each long-term condition among trans and non-binary respondents and all other survey respondents are presented in Table 2, with 39.3% of trans and non-binary respondents and 38.3% of all other respondents reporting no long-term health conditions. However, after adjustment for age, ethnicity and deprivation, overall, for 10 out of the 15 long-term conditions, trans and non-binary adults reported higher prevalence, with inequalities largest for autism, OR (95%CI), 5.8 (5.0 - 6.6), dementia, 3.1 (2.5 - 3.9), learning disabilities, 2.8 (2.4 - 3.2) and mental health, 2.0 (1.9 - 2.2) (Table 2).   Trans and non-binary respondents were 30% less likely to report no long-term conditions after adjustment for age, deprivation and ethnicity, 0.7 (0.7 - 0.8).

<Insert Table 2 about here>

For peer review only

Table 2. Long-term health conditions (735,078 responses, 5110 trans and non-binary)

	All trans and non-binary respondents (n, weighted %)	All other survey respondents (n, weighted %)	Adjusted for age, deprivation and ethnicity OR (95%CI)	p-value
Autism or autism spectrum condition	227 (4.4)	4236 (0.6)	5.8 (5.0 - 6.6)	<0.0001
Alzheimer's disease or other cause of dementia	96 (1.9)	5471 (0.7)	3.1 (2.5 - 3.9)	<0.0001
A learning disability	204 (4.0)	7607 (1.0)	2.8 (2.4 - 3.2)	<0.0001
A mental health condition	891 (17.4)	66363 (9.1)	2.0 (1.9 - 2.2)	<0.0001
A stroke (which affects your day to day life)	81 (1.6)	7638 (1.0)	1.8 (1.4 - 2.2)	<0.0001
Blindness or partial sight	118 (2.3)	11955 (1.6)	1.7 (1.4 - 2.0)	<0.0001
Kidney or liver disease	156 (3.1)	17038 (2.3)	1.4 (1.2 - 1.6)	<0.0001
A neurological condition such as epilepsy	116 (2.3)	13855 (1.9)	1.3 (1.1 - 1.6)	0.003
Deafness or hearing loss	287 (5.6)	55229 (7.6)	1.2 (1.1 - 1.4)	0.002
Diabetes	657 (12.9)	72176 (9.9)	1.2 (1.1 - 1.4)	<0.0001
Another long-term condition or disability	645 (12.6)	94872 (13.0)	1.1 (1.0 - 1.2)	0.009
A heart condition such as angina or atrial fibrillation	279 (5.5)	55841 (7.6)	1.0 (0.9 - 1.2)	0.74
A breathing condition such as asthma or COPD	523 (10.2)	89611 (12.3)	1.0 (0.9 - 1.0)	0.28
Arthritis or ongoing problem with back or joints	865 (16.9)	166498 (22.8)	0.9 (0.9 - 1.0)	0.18
High blood pressure	867 (17.0)	158434 (21.7)	0.9 (0.9 - 1.0)	0.055
Cancer (diagnosis or treatment in the last 5 years)	121 (2.4)	31555 (4.3)	0.9 (0.7 - 1.0)	0.092
I do not have any long term conditions	2006 (39.3)	279314 (38.3)	0.7 (0.7 - 0.8)	<0.0001

For peer review only

Adjusted percentages stratified by age are presented in Figure 1 and Appendix table 2 for model estimates; in general the differences between trans and non-binary adults (black lines) and all other survey respondents (grey lines) are smaller than the differences between conditions. Trans and non-binary adults are presenting to primary care with similar health profiles to those of all other survey respondents; the age related patterns of long term conditions and variation between conditions is similar across both trans and non-binary respondents and all other survey respondents.

<Insert Figure 1 about here>

Figure 1. Long term condition prevalence, stratified by age. Black line - all trans and/or non-binary respondents; Grey - all other survey respondents

However, despite these over-arching similarities in prevalence, on inspection, there are four different groups of age related patterns across conditions. First, both autism and autistic spectrum conditions, and MH problems, have higher prevalence at younger ages, and prevalence among trans and non-binary respondents are particularly high at younger ages with absolute differences reducing with age. Second, for dementia, learning disability, blindness, and neurological conditions including epilepsy, adjusted prevalence in trans and non-binary adults follows the same pattern as all other survey respondents, but prevalence shifted upwards with higher prevalence at each age, although absolute differences are small. Third, for stroke, diabetes, and kidney or liver problems the disparities increase with age, with trans and non-binary adults having increasingly higher prevalence compared with all other survey respondents at older age. Finally, for arthritis, hypertension, cancer, breathing problems, heart conditions and deafness, adjusted prevalence in trans and non-binary adults at older ages is lower than prevalence for all other survey respondents (Figure 1, Appendix table 2 for model estimates and Appendix figures 1-3 for versions of Figure 1 with rescaled axes for clarity).

Patient experience

After adjusting for age, ethnicity, deprivation and practice we found that trans and non-binary adults reported slightly poorer overall primary care experiences than all other survey respondents (Table 3) 0.8 (0.8 - 0.9); and across access and overall experience items reported higher numbers of both very good and very poor experiences (Table 4), however there were stronger differences across different domains of patient experience.

<Insert Tables 3 and 4 about here>

For peer review only

Table 3. Patient experience, coding for public reporting

Experience items	Number of responses	All trans and non-binary respondents (n, weighted % positive response)	All other survey respondents (n, weighted % positive response)	OR (95% CI) adjusted for age, deprivation, ethnicity	p-value
**Overall experience**					
Overall experience	816,702	80.3	85.7	0.8 (0.8 - 0.9)	<0.0001
Overall experience of making an appointment	752,868	71.4	73.9	1.0 (1.0 - 1.1)	0.84
**Before trying to make an appointment**					
Tried self-management	*742,768*	*30.4*	*31.7*	*0.9 (0.8 - 0.9)*	*<0.0001*
Asked friends or family	*742,768*	*8.4*	*7.7*	*1.0 (0.9 - 1.1)*	*0.53*
Tried online, telephone or other NHS services	*742,768*	*25.0*	*16.8*	*1.3 (1.3 - 1.4)*	*<0.0001*
Tried online or other non-NHS services	*742,768*	*51.8*	*58.0*	*0.9 (0.8 - 0.9)*	*<0.0001*
**Access**					
Found GP practice website easy to use	389,957	70.9	77.1	0.8 (0.8 - 0.9)	<0.0001
Tried to make an appointment in the last 6 months	*777,102*	*66.5*	*64.8*	*1.0 (1.0 - 1.1)*	*0.69*
Getting through on the phone	789,116	69.7	72.5	1.0 (0.9 - 1.1)	0.82
Helpful receptionists	795,619	86.9	90.9	0.9 (0.8 - 0.9)	0.0001
Offered choices when booking appointment	570,565	74.9	71.4	1.2 (1.2 - 1.3)	<0.0001
Satisfied with appointment times available	715,175	73.0	71.0	1.2 (1.1 - 1.3)	<0.0001
Offered an acceptable appointment	749,975	87.4	90.7	0.8 (0.7 - 0.9)	<0.0001
Satisfied with appointment offered	695,269	79.8	83.5	1.0 (0.9 - 1.0)	0.18
Remote appointment (telephone or online)	648,288	53.9	49.2	1.1 (1.1 - 1.2)	<0.0001
**Continuity**					
Have a preferred GP	*776,310*	*64.2*	*50.2*	*1.7 (1.6 - 1.8)*	*<0.0001*
Able to see preferred GP	216,333	59.5	58.8	1.1 (1.0 - 1.2)	0.12
**Communication**					
Involved in decisions about care and treatment	668,530	85.0	93.7	0.5 (0.5 - 0.6)	<0.0001
Had MH needs in last appointment	*604,671*	*76.2*	*55.5*	*2.1 (1.9 - 2.2)*	*<0.0001*
MH needs recognised and understood	336,574	77.6	87.3	0.7 (0.6 - 0.7)	<0.0001
Confidence and trust	744,575	88.7	96.3	0.4 (0.4 - 0.4)	<0.0001
Needs were met	745,767	88.1	95.2	0.5 (0.5 - 0.5)	<0.0001

Experience items marked in *italics* refer to preferences or choices, rather than evaluation of care quality. These questions as well as evaluative items are presented side by side here for clarity across the patient journey

For peer review only

Table 4. Patient experience - very positive and very negative responses

	Adjusted % endorsing the most positive response option		Adjusted % endorsing the most negative response option	
	Trans and non-binary respondents	All other survey respondents	Trans and non-binary respondents	All other survey respondents
**Overall experience**				
Overall experience	54.4 (53.1 - 55.6)	53.3 (53.2 - 53.5)	3.3 (2.9 - 3.7)	1.9 (1.8 - 1.9)
Overall experience of making an appointment	41.2 (39.8 - 42.5)	36.3 (36.2 - 36.4)	5.8 (5.2 - 6.4)	4.6 (4.5 - 4.6)
**Access**				
Found GP practice website easy to use	31.9 (30.3 - 33.5)	26.7 (26.6 - 26.8)	9.1 (8.2 - 10.1)	6.8 (6.7 - 6.9)
Getting through on the phone	33.7 (32.5 - 35.0)	25.3 (25.2 - 25.4)	9.8 (9.1 - 10.5)	9.5 (9.5 - 9.6)
Helpful receptionists	56.0 (54.7 - 57.2)	50.3 (50.2 - 50.4)	3.2 (2.8 - 3.6)	2.4 (2.3 - 2.4)
**Communication**				
Involved in decisions about care and treatment	60.4 (59.0 - 61.8)	64.0 (63.9 - 64.1)	11.5 (10.7 - 12.4)	6.3 (6.2 - 6.3)
Confidence and trust	68.3 (67.1 - 69.6)	74.1 (74.0 - 74.2)	8.7 (8.0 - 9.5)	3.7 (3.7 - 3.8)
Needs were met	63.7 (62.3 - 65.0)	67.7 (67.6 - 67.8)	9.1 (8.4 - 9.8)	4.9 (4.8 - 4.9)

For peer review only

There was no evidence of a difference in overall experience of making an appointment, 1.0 (1.0-1.1), with only small differences in experiences across different domains of access (OR range 0.8 to 1.2 across items), however for continuity, trans and non-binary adults were much more likely to report that they had a preference for a particular GP 1.7 (1.6 - 1.8), with no difference compared with all other survey respondents in whether they were able to see this preferred GP or not 1.1 (1.0 - 1.2). Disparities in experiences of interpersonal communication were greatest. After adjustment, trans and non-binary respondents reported experiences of communication about 15 percentage points poorer than all other survey respondents. Trans and non-binary respondents, more likely to report having MH needs during their appointment 2.1 (1.9 - 2.2), however, among people with MH needs, trans and non-binary people were less likely to report that these needs had been met (Table 3).

For peer review only

Discussion

Trans and non-binary adults in England responding to the GP Patient Survey reported poorer health and poorer experiences in primary care than all other survey respondents. This research adds up to date evidence in an area where our understanding of the population demographics and healthcare need is still emerging, from a large, nationally representative, sample. This analysis highlights that autism and autistic spectrum conditions, dementia, learning disability and mental health problems are the conditions where disparities are greatest, with variation by age. In healthcare experience, disparities are much greater in interpersonal communication than access. Additionally, trans and non-binary adults report much higher preference for continuity of care than all other survey respondents, with no evidence of any differences in being able to see or speak to a preferred GP. For measures of overall primary care experience, and access, both more very positive and more very negative experiences were reported.

These results, considering both health outcomes and patient experience in primary care, are particularly important in the context of evidence that healthcare outcomes among trans and gender diverse individuals are better when accessing a primary care provider who is knowledgeable about trans health.[15] They also provide insight for understanding the demographics of the trans population in primary care, and the health needs of this group.

Our finding that trans and non-binary adults are over-represented among adults in autism and autistic spectrum conditions, dementia, learning disability and mental health problems in primary care is consistent with previous research which found higher rates of autism, other neurodevelopmental and psychiatric diagnoses in trans and gender-diverse study participants.[16] Our findings of poorer healthcare experiences from trans and non-binary adults also reflect findings from earlier work.[2] Our analyses build on and develop the evidence base from these previous studies, with population-based nationally representative estimates, and provide additional insight about the size of these disparities for most long-term health conditions.

This work provides novel insight into how disparities in long-term health conditions among trans and non-binary individuals vary with age. The four different age-related patterns for long-term conditions point to heterogeneous and potentially intersecting factors driving some of the differences seen. It is interesting to note that this variation in age-related patterns between long-term health conditions is not seen when considering inequalities in long-term health conditions by socio-economic deprivation, for which inequalities consistently increase with age,[17] nor by sexual orientation, where disparities across almost all long-term health conditions are greatest at younger ages and decrease at older ages.[18] For trans and non-binary adults the drivers of differences are likely to be more condition-specific. These patterns are likely the result of complex interactions between minority stress, behavioural risk factors, and biological effects of exogenous hormones, as well as sexual orientation and socioeconomic status. In addition, trans and non-binary people are not a single homogenous group and the patterns observed in long term health conditions may be in part driven by differences across subgroups.

For peer review only

From an epidemiological perspective, it is likely that age, period and cohort effects may all be important in understanding these relationships. For example, the impact of legislation including the Local Government Act (Section 28) in 1988,[19] Gender Recognition Act in 2004,[20] and the Equalities Act in 2010[21] are likely to have had an impact for trans adults during these years (period effects). Mental health, may have been affected by external factors such as transphobic media focus, in more recent years. Long-term gender-affirming therapies may have specific health impacts over time (age effects), and we speculate but cannot confirm that this may be part of the explanation for differences in prevalence of some long-term conditions which are hormonally mediated. Higher mortality among trans and non-binary adults may potentially also have an impact on long-term condition prevalence at older ages, but this cannot be evaluated in this cross-sectional analysis.

We note that trans and non-binary survey respondents were much younger, on average than all other survey respondents, which may suggest that people are more comfortable coming out as trans and at younger ages than previously.[22] The experiences of people who are trans and in their late teens / early 20s in 2021 may be very different from a similarly aged cohort in the past. The much higher prevalence of autism and autistic spectrum conditions and mental health problems among young trans adults compared with older trans adults may reflect these cohort effects as well. Longitudinal population-based data collections are needed to properly disentangle the impacts of coming out at different ages on our understanding of these health impacts.[23]

Although the patterns of age-specific long-term health condition prevalence identified provide some insight into disparities experienced by trans and non-binary adults, it is also important to highlight that for most conditions differences in patterns of prevalence between trans and non-binary and all other survey respondents are smaller than differences in prevalence between conditions. This finding highlights that for long-term condition management in primary care the epidemiology of conditions among trans and non-binary adults within a practice is likely to be similar to all other patients within the practice. Standard best practice for the management of long-term conditions, unrelated to trans status, will likely form a large part of the work of GPs with trans and non-binary patients in their practice. Previous work has highlighted that guidelines beyond transition or HIV care still need development.[9] Research areas that underpin long-term condition management, such as ensuring risk prediction models for long-term conditions can be correctly implemented for trans and non-binary patients will remain a priority.[24]

The areas where there are substantial differences in prevalence, however, are for autism and autistic spectrum conditions, and mental health problems, which are both very high among young trans and non-binary adults. In 2016 the Royal College of General Practitioners produced a position statement and best practice guidance, making commitments to enabling access for patients on the autistic spectrum to primary care, and recognising the additional needs of those with autism.[25] Similar guidance for mental health problems was published in 2017.[26] These guidelines are approaches which, again although not trans-specific, provide practical strategies which may be worth considering for addressing disparities in the poorer healthcare experiences of trans and non-binary patients in primary care.

For peer review only

Our findings describing the patient experience of trans and non-binary patients in primary care provide further insight into where quality improvement work could be directed. The relatively small disparities in access compared with the much larger inequalities in communication experienced by trans and non-binary adults are one such example. Resources are beginning to be developed to support best practice for primary care for communication with trans and non-binary adults in primary care. The 'healthtalk' resource includes interviews with 50 trans and gender diverse young people highlighting individual reflections on how experiences of health care could be improved.[27] Collections of individual experiences provide important insight where evidence-based best practice guidelines are still developing.

The much higher number of trans and non-binary patients who have a preference for continuity is also worth highlighting. Some GPs are regarded by the trans community as providing good trans healthcare and therefore more trans people may register with these GPs[28] whilst surgeries where care is poorer may lose their trans patients. Some trans and non-binary people report having excellent GPs with high levels of training and understanding of trans patients while many do not; this may explain our finding of both very positive and very negative experiences. Our findings that trans and non-binary adults in England are more likely to be living with long-term health conditions, again highlights the importance of continuity.[29] It also highlights that all GPs, regardless of location or special interest are likely to encounter trans and non-binary patients and will be supporting particular needs relating to long term health conditions. Where evidence for this work is lacking further dedicated studies are warranted, with sufficient funding and co-production with the trans and non-binary community.

It is important to identify that there are limitations to the findings from this research; several of these have been discussed in the protocol.[14] From a survey methods perspective, non-differential misclassification of trans and non-binary adults is one concern which has been recognised recently in the development of tools to collect gender and trans status in the NATSAL 4 study.[30,31] Although the number of people incorrectly reporting a trans or non-binary identity will be small, they may form a non-trivial number of trans and non-binary respondents overall. This is one possible explanation for the association seen with dementia, where numbers are low, and inaccuracies may be higher. However the consistency of the findings from this work with previous studies where there were interviewer-led surveys (including, for example, the finding of higher endorsement of both very good and very poor patient experience items)[32] give some reassurance that although this is a concern the impact may be low. A second limitation is the exclusion of respondents who didn't respond, or responded "prefer not to say" to questions about gender and trans-status, it is a concern that these responses may differentially include people who are trans and non-binary. Previous analyses of the GP Patient Survey focusing on sexual orientation found poorer experiences in these groups.[33] It is also worth highlighting that these findings are limited to trans and non-binary patients who are registered with a GP, and the population who are not registered, or who access primary care only through private GP services such as Babylon,[28] will not be included.

For peer review only

Despite these limitations, the collection of trans status and an inclusive gender question in the GP Patient Survey is an important step towards the generation of evidence for the development of best practice guidance for trans and non-binary patients. Following guidance on delivering good care and communication for those with autism and autistic spectrum conditions, and mental health problems, both of which are prevalent among young trans and non-binary adults, is one way some of the inequalities identified may be addressed. Long-term condition epidemiology, although with some variation between conditions, is broadly similar in both trans and non-binary adults, and all other survey respondents. Good primary care, not just good trans healthcare, is important to address the disparities identified.

For peer review only

Funding

C.L.S. holds a Career Development Fellowship (award number: MH041) funded as part of the Three NIHR Research Schools Mental Health Programme [*]. A.M.M. is funded by the EU/EFPIA Innovative Medicines Initiative Joint Undertaking BigData@Heart grant 116074. This research was supported by core funding from the: British Heart Foundation (RG/13/13/30194; RG/18/13/33946), and NIHR Cambridge Biomedical Research Centre (BRC-1215-20014) [*]. * The views expressed are those of the author(s) and not necessarily those of the NIHR or the Department of Health and Social Care. J.L.'s work for this study is supported by the Wellcome Trust as part of the Wellcome Trust PhD programme for Primary Care Clinicans [grant number RHZB/a95].

Ethical approval

This is a secondary analysis of anonymised routinely collected data; formal ethical approval is not required

Competing interests

None

Data availability

Person-level data cannot be shared but the tool on the website www.gp-patient.co.uk is excellent for interested researchers to explore the data further

Contributorship Statement

CLS carried out the analysis, wrote the first draft of the paper and is guarantor. CLS, JL, AMM, MR, JS and RD developed the analysis protocol. All authors read, critically revised and reviewed the final draft.

For peer review only - http://bmjopen.bmj.com/site/about/guidelines.xhtml

References

1. Ellis SJ, Bailey L, McNeil J. Trans People's Experiences of Mental Health and Gender Identity Services: A UK Study. Journal of Gay & Lesbian Mental Health 2015;19:4-20.

2. Government Equalities Office. National LGBT Survey Research Report. https://www.gov.uk/government/organisations/government-equalities-office. 2018.

3. Stonewall. LGBT in Britain - Trans report https://www.stonewall.org.uk/lgbt-britain-trans-report. 2018.

4. Stonewall. LGBT in Britain - Health https://www.stonewall.org.uk/lgbt-britain-health. 2018.

5. Ipsos MORI, NHS England. GP Patient Survey National Report 2021 Publication https://www.gp-patient.co.uk/surveysandreports. 2021.

6. TransActual. Trans lives survey 2021 https://www.transactual.org.uk/trans-lives-21. 2021.

7. NHS England. The NHS Long Term Plan. https://www.longtermplan.nhs.uk/wp-content/uploads/2019/08/nhs-long-term-plan-version-1.2.pdf. 2019.

8. Ford J, Sowden S, Olivera J, et al. Transforming health systems to reduce health inequalities. Future Healthc J 2021;8:e204-e9.

9. Dahlen S, Connolly D, Arif I, Junejo MH, Bewley S, Meads C. International clinical practice guidelines for gender minority/trans people: systematic review and quality assessment. BMJ Open 2021;11:e048943.

10. Berner AM, Connolly DJ, Pinnell I, et al. Attitudes of transgender men and non-binary people to cervical screening: a cross-sectional mixed-methods study in the UK. Br J Gen Pract 2021;71:e614-e25.

11. Ipsos MORI, NHS. GP Patient Survey 2021 Technical Annex https://www.gp-patient.co.uk/downloads/2021/GPPS_2021_Technical_Annex_PUBLIC.pdf. 2021.

12. Office for National Statistics. Gender identity https://www.ons.gov.uk/census/censustransformationprogramme/questiondevelopment/genderidentity. 2020.

13. Ipsos MORI, NHS. GP Patient Survey - Questionnaire https://www.gp-patient.co.uk/downloads/2021/qandletter/GPPS_2021_Questionnaire_PUBLIC.pdf. 2021.

14. Saunders CL, Lund J, Mason AM, Roberts M, Smith J, Duschinsky R. The Health and Healthcare Outcomes of Trans and/or Non-Binary Adults in England: Protocol for an Analysis of Responses to the 2021 GP Patient Survey. Sexes 2022;3:325-35.

15. Kattari SK, Call J, Holloway BT, Kattari L, Seelman KL. Exploring the Experiences of Transgender and Gender Diverse Adults in Accessing a Trans Knowledgeable Primary Care Physician. Int J Environ Res Public Health 2021;18.

16. Warrier V, Greenberg DM, Weir E, et al. Elevated rates of autism, other neurodevelopmental and psychiatric diagnoses, and autistic traits in transgender and gender-diverse individuals. Nat Commun 2020;11:3959.

17. Barnett K, Mercer SW, Norbury M, Watt G, Wyke S, Guthrie B. Epidemiology of multimorbidity and implications for health care, research, and medical education: a cross-sectional study. Lancet 2012;380:37-43.

18. Saunders CL, MacCarthy S, Meads C, et al. Long-term conditions among sexual minority adults in England: Evidence from a cross-sectional analysis of responses to the English General Practice Patient Survey. BJGP Open 2021;DOI: 10.3399/BJGPO.2021.0067.

19. HM Government. Local Government Act https://www.legislation.gov.uk/ukpga/1988/9/contents. 1988.

For peer review only

20. HM Government. Gender Recognition Act https://www.legislation.gov.uk/ukpga/2004/7/contents. 2004.

21. HM Government. Equality Act https://www.legislation.gov.uk/ukpga/2010/15/contents. 2010.

22. Zhang Q, Goodman M, Adams N, et al. Epidemiological considerations in transgender health: A systematic review with focus on higher quality data. Int J Transgend Health 2020;21(2):125-37

23. MacCarthy S, Saunders CL, Elliott MN. Increased Reporting of Sexual Minority Orientation from 2009 to 2017 in England and Implications for Measuring Sexual Minority Health Disparities. LGBT Health 2020;7:393-400.

24. Berner AM, Lund J, Saunders CL. Tackling the complexity of gender bias in primary care. Br J Gen Pract 2021;71:296-7.

25. Royal College of General Practitioners. RCGP Position Statement on Autistic Spectrum Disorders https://www.rcgp.org.uk/policy/rcgp-policy-areas/autistic-spectrum-disorders.aspx. 2016.

26. Royal College of General Practitioners. RCGP Position Statement on Mental Health in Primary Care https://www.rcgp.org.uk/policy/rcgp-policy-areas/mental-health-in-primary-care.aspx. 2017.

27. healthtalk.org. Experiences of trans and gender diverse young people: GP surgery experiences https://healthtalk.org/Experiences-of-trans-and-gender-diverse-young-people/Trans-and-gender-diverse-young-peoples-experience-of-GP-surgeries. 2022.

28. Action for Trans Health. List of trans friendly GPs https://actionfortranshealth.org.uk/resources/for-trans-people/list-of-trans-friendly-gps/ 2021.

29. Paddison CAM, Rosen R. Tackling the crisis in primary care. Bmj 2022;377:o1485.

30. Connolly A. Presentation at the Taiwan-UK SGS Health Research Network July 17th 2022. 2022.

31. NatCen. Question testing for the National Survey of Sexual Attitudes and Lifestyles 4. Report on findings from cognitive interviews https://www.natsal.ac.uk/sites/default/files/2020-11/Natsal-4-Cognitive-Testing-Report-2019_Publication-1.pdf. 2019.

32. Yarrow E. Gendered bodies: the experiences of gender and sex diverse children and youth in health and education institutions, Centre for Gender Studies, University of Cambridge. 2017.

33. Elliott MN, Kanouse DE, Burkhart Q, et al. Sexual minorities in England have poorer health and worse health care experiences: a national survey. J Gen Intern Med 2015;30(1):9-16

For peer review only

For peer review only

For peer review only

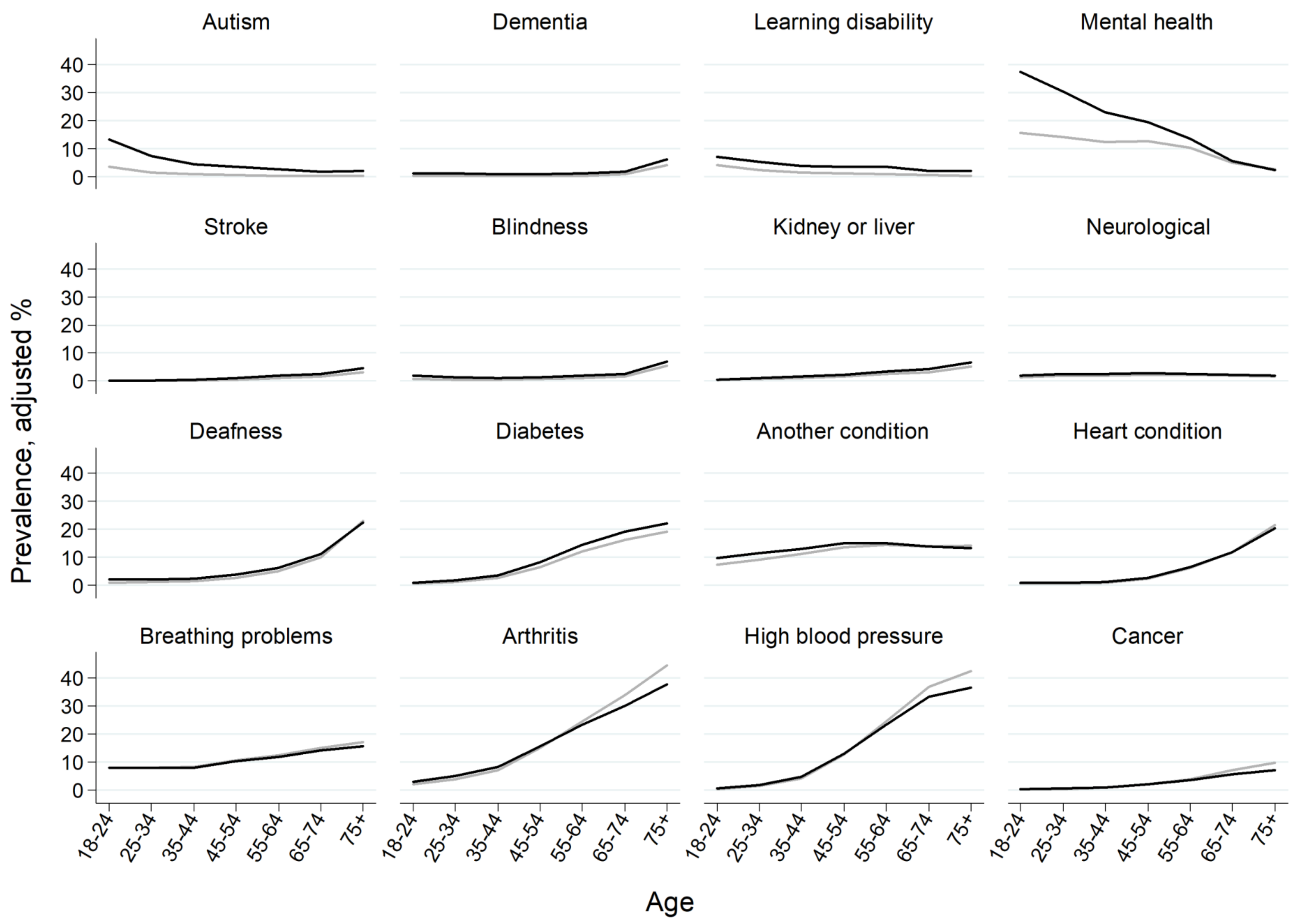
Autism
Dementia
Learning disability
Mental health
Stroke
Blindness
Kidney or liver
Neurological
Deafness
Diabetes
Another condition
Heart condition
Breathing problems
Arthritis
High blood pressure
Cancer
Prevalence, adjusted %
0
10
20
30
40
18-24
25-34
35-44
45-54
55-64
65-74
75+
Age

Appendix Table 1. Respondent characteristics; column percentages

	All trans and non-binary respondents (n, column %)	All other survey respondents (n, column %)
**Gender**		
Female	1708 (28.2)	468958 (56.5)
Male	1971 (32.6)	359266 (43.3)
Non-binary	1220 (20.2)	
Prefer to self-describe	1047 (17.3)	
Prefer not to say	103 (1.7)	1288 (0.2)
**Trans status**		
Gender identity the same as sex registered at birth	957 (15.3)	825209 (99.6)
Gender identity different from sex registered at birth	4642 (74.4)	
Prefer not to say	644 (10.3)	3294 (0.4)
**Age**		
16-24	628 (10.1)	41162 (5.0)
25-34	838 (13.5)	69764 (8.4)
35-44	1109 (17.9)	100108 (12.1)
45-54	1155 (18.6)	137231 (16.6)
55-64	1080 (17.4)	174512 (21.1)
65-74	827 (13.3)	172155 (20.8)
75-84	573 (9.2)	132384 (16.0)
**Ethnicity**		
White	3343 (54.0)	702888 (84.9)
Asian	176 (2.8)	10780 (1.3)
Black	1467 (23.7)	70305 (8.5)
Mixed	482 (7.8)	27612 (3.3)
Other	727 (11.7)	16481 (2.0)
**Sexual orientation**		
Heterosexual	2682 (46.1)	754746 (92.7)
Lesbian / gay	310 (5.3)	11452 (1.4)
Bisexual	462 (7.9)	8186 (1.0)
Other	752 (12.9)	6235 (0.8)
Prefer not to say	1614 (27.7)	33674 (4.1)
**Deprivation**		
Most deprived	2211 (34.9)	162804 (19.5)
2	1650 (26.1)	167119 (20.0)
3	1117 (17.6)	172925 (20.7)
4	791 (12.5)	169938 (20.4)
Least deprived	561 (8.9)	161387 (19.3)

For peer review only

Appendix table 2. All age stratified OR and 95%CI (from the model underpinning Figure 1)

	16-24	25-34	35-44	45-54	55-64	65-74	75+
Autism or autism spectrum condition	4.4 (3.4 - 5.6)	5.5 (4.1 - 7.3)	5.4 (3.7 - 7.8)	7.1 (4.9 - 10.3)	10.5 (7.0 - 15.7)	10.5 (5.6 - 20.0)	8.0 (3.3 - 19.6)
Alzheimer's disease or other cause of dementia	2.7 (0.4 - 20.2)	15.2 (8.1 - 28.6)	12.7 (7.5 - 21.4)	6.6 (3.6 - 11.8)	4.7 (2.7 - 8.1)	2.9 (1.7 - 4.8)	1.3 (0.8 - 1.9)
A learning disability	1.9 (1.4 - 2.7)	2.2 (1.6 - 3.0)	2.5 (1.7 - 3.6)	3.7 (2.6 - 5.1)	4.0 (2.8 - 5.8)	7.1 (4.5 - 11.4)	4.6 (2.0 - 10.3)
A mental health condition	4.4 (3.7 - 5.3)	3.0 (2.6 - 3.6)	1.2 (1.0 - 1.5)	1.4 (1.2 - 1.7)	1.5 (1.2 - 1.8)	1.8 (1.4 - 2.4)	1.8 (1.1 - 2.9)
A stroke (which affects your day to day life)	1.6 (1.0 - 2.4)	3.4 (1.1 - 10.7)	3.2 (1.5 - 6.8)	2.2 (1.3 - 3.8)	1.2 (0.7 - 2.1)	2.0 (1.3 - 3.1)	1.6 (1.0 - 2.4)
Blindness or partial sight	2.8 (1.5 - 5.3)	2.5 (1.3 - 4.8)	1.8 (1.0 - 3.4)	2.2 (1.4 - 3.5)	1.6 (1.0 - 2.5)	1.7 (1.1 - 2.6)	1.1 (0.7 - 1.6)
Kidney or liver disease	2.0 (0.7 - 5.4)	0.7 (0.3 - 1.9)	2.0 (1.3 - 3.0)	1.4 (1.0 - 2.1)	1.3 (0.9 - 1.9)	1.1 (0.7 - 1.6)	1.5 (1.0 - 2.1)
A neurological condition such as epilepsy	1.3 (0.6 - 2.5)	2.1 (1.4 - 3.2)	0.9 (0.5 - 1.5)	1.5 (1.0 - 2.2)	1.1 (0.7 - 1.7)	1.5 (0.9 - 2.4)	0.9 (0.4 - 2.0)
Deafness or hearing loss	1.1 (0.5 - 2.5)	2.5 (1.5 - 4.0)	1.9 (1.3 - 2.9)	1.4 (1.0 - 2.0)	1.4 (1.0 - 1.8)	1.1 (0.9 - 1.5)	0.9 (0.7 - 1.1)
Diabetes	0.2 (0.0 - 1.7)	1.2 (0.8 - 2.0)	1.5 (1.1 - 1.9)	1.4 (1.1 - 1.7)	1.1 (0.9 - 1.3)	1.3 (1.1 - 1.6)	1.2 (0.9 - 1.4)
Another long-term condition or disability	1.8 (1.4 - 2.3)	1.7 (1.3 - 2.1)	1.0 (0.8 - 1.2)	0.8 (0.6 - 0.9)	1.1 (0.9 - 1.3)	1.1 (0.9 - 1.4)	1.3 (1.0 - 1.7)
A heart condition such as angina or atrial fibrillation	1.6 (0.6 - 3.8)	1.3 (0.6 - 2.8)	1.2 (0.7 - 2.2)	1.0 (0.6 - 1.5)	1.1 (0.9 - 1.4)	1.0 (0.8 - 1.3)	0.9 (0.7 - 1.2)
A breathing condition such as asthma or COPD	1.2 (0.9 - 1.6)	1.0 (0.8 - 1.3)	0.8 (0.6 - 1.1)	1.0 (0.8 - 1.2)	0.9 (0.7 - 1.1)	1.0 (0.8 - 1.2)	1.0 (0.7 - 1.3)
Arthritis or ongoing problem with back or joints	2.5 (1.7 - 3.7)	1.4 (1.1 - 2.0)	1.3 (1.0 - 1.6)	0.8 (0.7 - 1.0)	0.8 (0.7 - 1.0)	0.8 (0.7 - 1.0)	1.0 (0.8 - 1.2)
High blood pressure	1.3 (0.4 - 4.0)	1.4 (0.8 - 2.3)	1.3 (1.0 - 1.7)	1.0 (0.8 - 1.2)	0.9 (0.7 - 1.0)	0.9 (0.8 - 1.0)	0.8 (0.7 - 1.0)
Cancer (diagnosis or treatment in the last 5 years)	2.4 (0.6 - 9.8)	1.1 (0.4 - 3.5)	1.3 (0.7 - 2.5)	0.7 (0.4 - 1.2)	1.1 (0.8 - 1.5)	0.7 (0.5 - 1.0)	0.8 (0.5 - 1.1)
I do not have any long term conditions	0.3 (0.2 - 0.3)	0.5 (0.4 - 0.6)	0.8 (0.7 - 1.0)	0.9 (0.8 - 1.0)	0.9 (0.8 - 1.0)	0.9 (0.8 - 1.1)	1.2 (0.9 - 1.6)

For peer review only

Appendix figure 1. Dementia, learning disability, blindness and neurological conditions. Long-term condition adjusted prevalence in trans and non-binary adults follows the same pattern as all other survey respondents, but prevalence shifted upwards with higher prevalence at each age

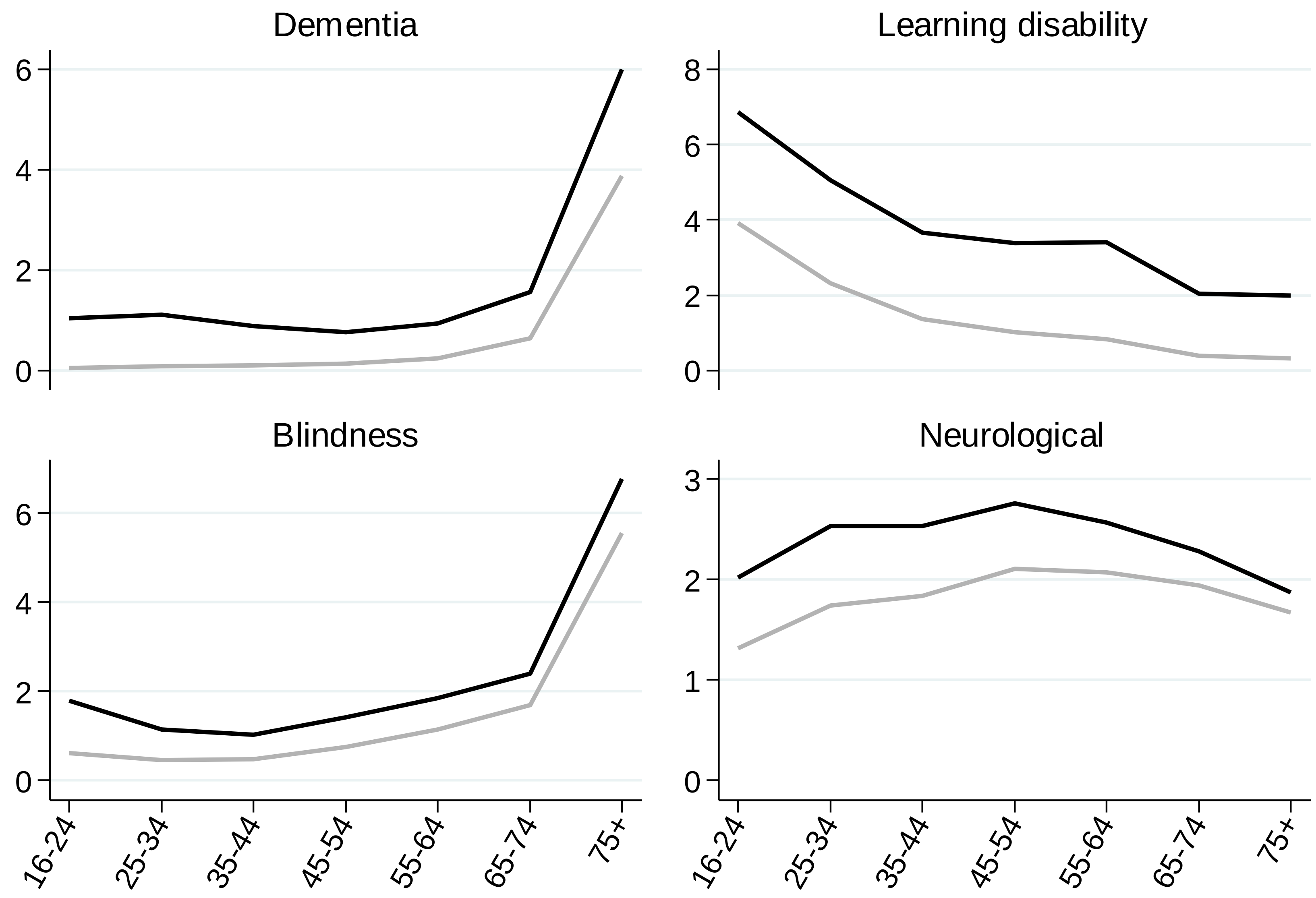

Appendix figure 2. Stroke, kidney or liver problems and diabetes; disparities increase with age, with trans and non-binary adults having increasingly higher prevalence compared with all other survey respondents at older age

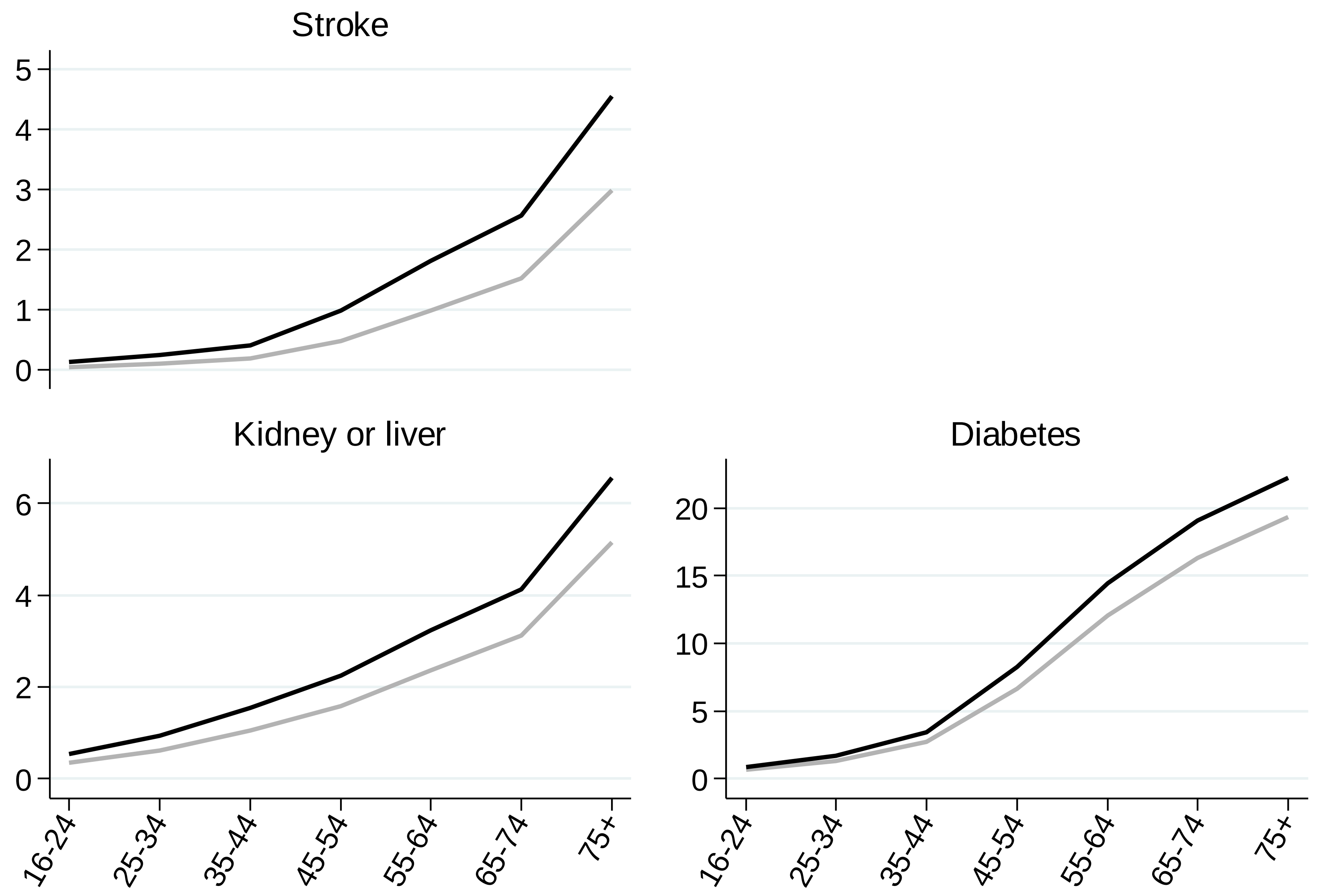

Appendix figure 3. Deafness, heart conditions, breathing problems, high blood pressure and cancer; adjusted prevalence in trans and non-binary adults at older ages is lower than prevalence for all other survey respondents

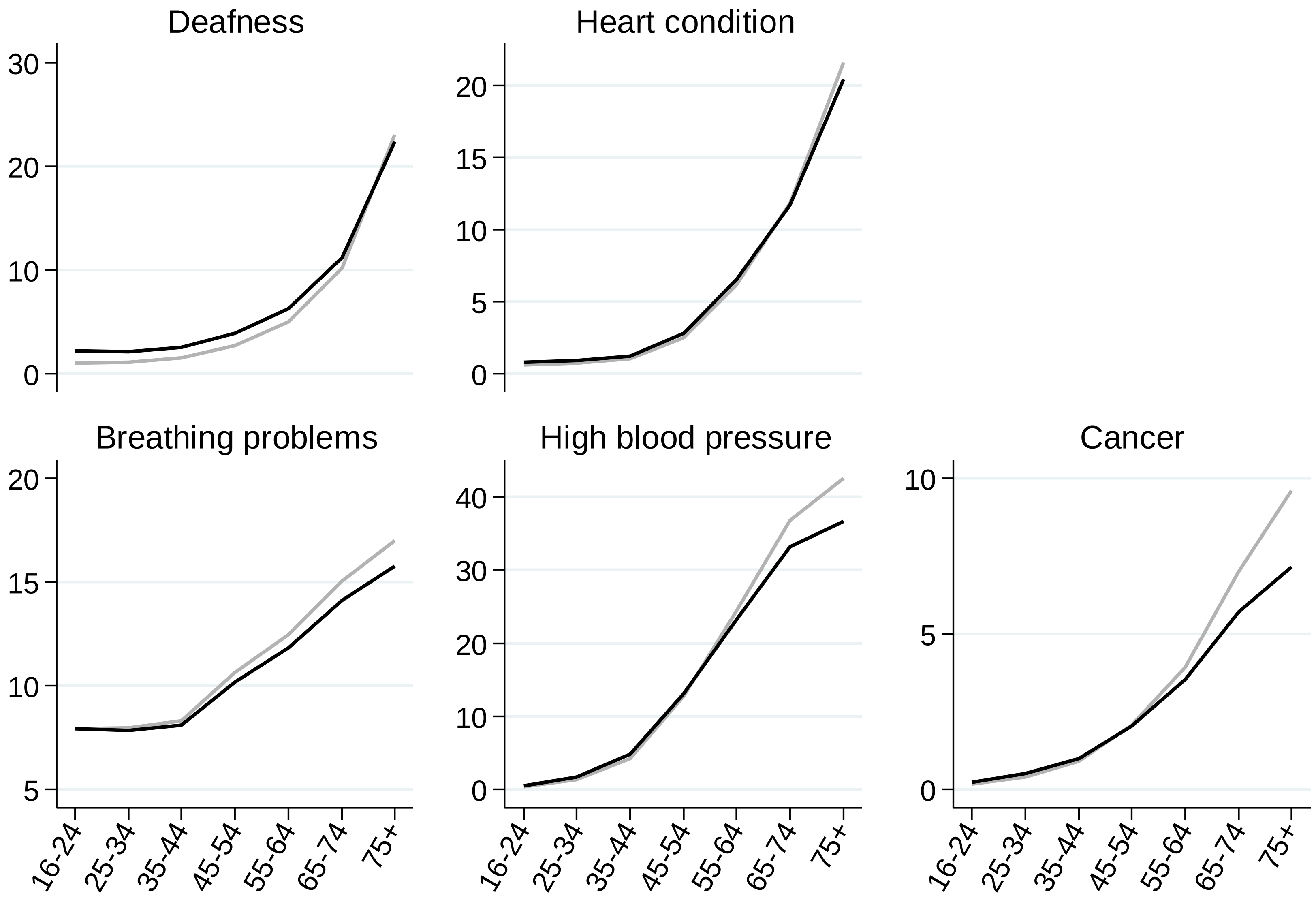

The RECORD statement – checklist of items, extended from the STROBE statement, that should be reported in observational studies using routinely collected health data.

	Item No.	STROBE items	Location in manuscript where items are reported	RECORD items	Location in manuscript where items are reported
**Title and abstract**					
	1	(a) Indicate the study's design with a commonly used term in the title or the abstract (b) Provide in the abstract an informative and balanced summary of what was done and what was found		RECORD 1.1: The type of data used should be specified in the title or abstract. When possible, the name of the databases used should be included.<br><br>RECORD 1.2: If applicable, the geographic region and timeframe within which the study took place should be reported in the title or abstract.<br><br>RECORD 1.3: If linkage between databases was conducted for the study, this should be clearly stated in the title or abstract.	p1, p2
**Introduction**					
Background rationale	2	Explain the scientific background and rationale for the investigation being reported			p4
Objectives	3	State specific objectives, including any prespecified hypotheses			p4
**Methods**					
Study Design	4	Present key elements of study design early in the paper			p5, p6
Setting	5	Describe the setting, locations, and relevant dates, including periods of recruitment, exposure, follow-up, and data collection			p5, plus pre-registered protocol

For peer review only

Participants	6	*(a) Cohort study* - Give the eligibility criteria, and the sources and methods of selection of participants. Describe methods of follow-up<br>*Case-control study* - Give the eligibility criteria, and the sources and methods of case ascertainment and control selection. Give the rationale for the choice of cases and controls<br>*Cross-sectional study* - Give the eligibility criteria, and the sources and methods of selection of participants<br><br>*(b) Cohort study* - For matched studies, give matching criteria and number of exposed and unexposed<br>*Case-control study* - For matched studies, give matching criteria and the number of controls per case		RECORD 6.1: The methods of study population selection (such as codes or algorithms used to identify subjects) should be listed in detail. If this is not possible, an explanation should be provided.<br><br>RECORD 6.2: Any validation studies of the codes or algorithms used to select the population should be referenced. If validation was conducted for this study and not published elsewhere, detailed methods and results should be provided.<br><br>RECORD 6.3: If the study involved linkage of databases, consider use of a flow diagram or other graphical display to demonstrate the data linkage process, including the number of individuals with linked data at each stage.	p5, plus pre-registered protocol
Variables	7	Clearly define all outcomes, exposures, predictors, potential confounders, and effect modifiers. Give diagnostic criteria, if applicable.		RECORD 7.1: A complete list of codes and algorithms used to classify exposures, outcomes, confounders, and effect modifiers should be provided. If these cannot be reported, an explanation should be provided.	n/a
Data sources/ measurement	8	For each variable of interest, give sources of data and details of methods of assessment (measurement).<br>Describe comparability of assessment methods if there is more than one group			p5, plus pre-registered protocol

For peer review only

Bias	9	Describe any efforts to address potential sources of bias			p5, plus details in pre-registered protocol
Study size	10	Explain how the study size was arrived at			p5, plus details in pre-registered protocol
Quantitative variables	11	Explain how quantitative variables were handled in the analyses. If applicable, describe which groupings were chosen, and why			p5, plus details in pre-registered protocol<br><br>(particularly for coding of patient experience items)
Statistical methods	12	(a) Describe all statistical methods, including those used to control for confounding<br>(b) Describe any methods used to examine subgroups and interactions<br>(c) Explain how missing data were addressed<br>(d) *Cohort study* - If applicable, explain how loss to follow-up was addressed<br>*Case-control study* - If applicable, explain how matching of cases and controls was addressed<br>*Cross-sectional study* - If applicable, describe analytical methods taking account of sampling strategy<br>(e) Describe any sensitivity analyses			p6
Data access and cleaning methods		..		RECORD 12.1: Authors should describe the extent to which the investigators had access to the database	P5, plus details in pre-registered protocol

For peer review only

				population used to create the study population.<br><br>RECORD 12.2: Authors should provide information on the data cleaning methods used in the study.	Authors had access to patient-level data under a data sharing agreement with NHS England
Linkage		..		RECORD 12.3: State whether the study included person-level, institutional-level, or other data linkage across two or more databases. The methods of linkage and methods of linkage quality evaluation should be provided.	No
**Results**					
Participants	13	(a) Report the numbers of individuals at each stage of the study (*e.g.*, numbers potentially eligible, examined for eligibility, confirmed eligible, included in the study, completing follow-up, and analysed)<br>(b) Give reasons for non-participation at each stage.<br>(c) Consider use of a flow diagram		RECORD 13.1: Describe in detail the selection of the persons included in the study (*i.e.,* study population selection) including filtering based on data quality, data availability and linkage. The selection of included persons can be described in the text and/or by means of the study flow diagram.	p7, Table 1
Descriptive data	14	(a) Give characteristics of study participants (*e.g.*, demographic, clinical, social) and information on exposures and potential confounders<br>(b) Indicate the number of participants with missing data for each variable of interest<br>(c) *Cohort study* - summarise follow-up time (*e.g.*, average and total amount)			p7, Table 1, Appendix Table 1

For peer review only

Outcome data	15	*Cohort study* - Report numbers of outcome events or summary measures over time<br>*Case-control study* - Report numbers in each exposure category, or summary measures of exposure<br>*Cross-sectional study* - Report numbers of outcome events or summary measures			Full details in Tables 2 and 3
Main results	16	(a) Give unadjusted estimates and, if applicable, confounder-adjusted estimates and their precision (e.g., 95% confidence interval). Make clear which confounders were adjusted for and why they were included<br>(b) Report category boundaries when continuous variables were categorized<br>(c) If relevant, consider translating estimates of relative risk into absolute risk for a meaningful time period			Tables 2-4 and appendix Table 2<br><br>Recycled predictions are presented for clarity
Other analyses	17	Report other analyses done—e.g., analyses of subgroups and interactions, and sensitivity analyses			p7, p8
**Discussion**					
Key results	18	Summarise key results with reference to study objectives			p9
Limitations	19	Discuss limitations of the study, taking into account sources of potential bias or imprecision. Discuss both direction and magnitude of any potential bias		RECORD 19.1: Discuss the implications of using data that were not created or collected to answer the specific research question(s). Include discussion of misclassification bias, unmeasured confounding, missing data, and changing eligibility over	p11

For peer review only

				time, as they pertain to the study being reported.	
Interpretation	20	Give a cautious overall interpretation of results considering objectives, limitations, multiplicity of analyses, results from similar studies, and other relevant evidence			p9, p10
Generalisability	21	Discuss the generalisability (external validity) of the study results			p11
**Other Information**					
Funding	22	Give the source of funding and the role of the funders for the present study and, if applicable, for the original study on which the present article is based			p13
Accessibility of protocol, raw data, and programming code		..		RECORD 22.1: Authors should provide information on how to access any supplemental information such as the study protocol, raw data, or programming code.	p13

*Reference: Benchimol EI, Smeeth L, Guttmann A, Harron K, Moher D, Petersen I, Sørensen HT, von Elm E, Langan SM, the RECORD Working Committee. The REporting of studies Conducted using Observational Routinely-collected health Data (RECORD) Statement. *PLoS Medicine* 2015; in press.

*Checklist is protected under Creative Commons Attribution (CC BY) license.

For peer review only - http://bmjopen.bmj.com/site/about/guidelines.xhtml

BMJ Open

Demographic characteristics, long-term health conditions, and healthcare experiences of 6,333 trans and non-binary adults in England: nationally representative evidence from the 2021 GP Patient Survey

Journal:	*BMJ Open*
Manuscript ID	bmjopen-2022-068099.R1
Article Type:	Original research
Date Submitted by the Author:	09-Dec-2022
Complete List of Authors:	Saunders, Catherine; University of Cambridge, Department of Public Health and Primary Care<br>Berner, Alison; Barts Cancer Institute; Tavistock and Portman NHS Foundation Trust<br>Lund, Jenny; University of Cambridge, Primary Care Unit, Department of Public Health & Primary Care<br>Mason, Amy M; University of Cambridge, Department of Public Health and Primary Care<br>Oakes-Monger, Tash; NHS England<br>Roberts, Meg; University of Cambridge<br>Smith, Jack; Lifestrong<br>Duschinsky, Robbie; University of Cambridge Primary Care Unit
**Primary Subject Heading**:	Health services research
Secondary Subject Heading:	General practice / Family practice
Keywords:	PRIMARY CARE, Quality in health care < HEALTH SERVICES ADMINISTRATION & MANAGEMENT, HEALTH SERVICES ADMINISTRATION & MANAGEMENT

SCHOLARONE™
Manuscripts

I, the Submitting Author has the right to grant and does grant on behalf of all authors of the Work (as defined in the below author licence), an exclusive licence and/or a non-exclusive licence for contributions from authors who are: i) UK Crown employees; ii) where BMJ has agreed a CC-BY licence shall apply, and/or iii) in accordance with the terms applicable for US Federal Government officers or employees acting as part of their official duties; on a worldwide, perpetual, irrevocable, royalty-free basis to BMJ Publishing Group Ltd ("BMJ") its licensees and where the relevant Journal is co-owned by BMJ to the co-owners of the Journal, to publish the Work in this journal and any other BMJ products and to exploit all rights, as set out in our licence.

The Submitting Author accepts and understands that any supply made under these terms is made by BMJ to the Submitting Author unless you are acting as an employee on behalf of your employer or a postgraduate student of an affiliated institution which is paying any applicable article publishing charge ("APC") for Open Access articles. Where the Submitting Author wishes to make the Work available on an Open Access basis (and intends to pay the relevant APC), the terms of reuse of such Open Access shall be governed by a Creative Commons licence – details of these licences and which Creative Commons licence will apply to this Work are set out in our licence referred to above.

Other than as permitted in any relevant BMJ Author's Self Archiving Policies, I confirm this Work has not been accepted for publication elsewhere, is not being considered for publication elsewhere and does not duplicate material already published. I confirm all authors consent to publication of this Work and authorise the granting of this licence.

For peer review only

Demographic characteristics, long-term health conditions, and healthcare experiences of 6,333 trans and non-binary adults in England: nationally representative evidence from the 2021 GP Patient Survey

Catherine L Saunders,[1,2*] Alison Berner,[3,4] Jenny Lund,[1] Amy M Mason,[5] Tash Oakes-Monger,[6] Meg Roberts,[7] Jack Smith,[8] Robbie Duschinsky[1]

1. Primary Care Unit, Department of Public Health and Primary Care, University of Cambridge, Cambridge, UK
2. Cambridge Public Health, University of Cambridge, Cambridge, UK
3. Barts Cancer Institute, Queen Mary University of London, London, UK
4. Gender Identity Clinic, Tavistock and Portman NHS Foundation Trust< London, UK
5. British Heart Foundation Cardiovascular Epidemiology Unit, Department of Public Health and Primary Care, University of Cambridge, Cambridge, UK
6. NHS England and NHS Improvement, London, UK
7. Newnham College, University of Cambridge, UK
8. Lifestrong, Wolverhampton, UK

*Corresponding author; ks659@medschl.cam.ac.uk

For peer review only

Abstract

Objective

In order to address the lack of data on the health and healthcare needs of trans and non-binary adults, NHS England includes questions asking about both gender and trans status in its surveys to support quality improvement programmes.

We used self-reported data from the GP Patient Survey to answer the research question: What are the demographic characteristics, health conditions, and healthcare experiences of trans and non-binary adults in England?

Design / Setting

Nationally representative, population-based cross-sectional survey in England with survey data collection from January-March 2021.

Participants

840,691 survey respondents including 6,333 trans and non-binary adults

Outcomes

We calculated weighted descriptive statistics, and using logistic regression explored 15 long-term physical and mental health conditions, and 18 patient experience items, covering overall experience, access, communication and continuity.

Results

Trans and non-binary adults were younger, more likely to be from Asian, Black, Mixed or Other ethnic groups, and more likely to live in more deprived parts of the country. Age-specific patterns of long-term conditions were broadly similar among trans and non-binary adults compared with all other survey respondents, with some variation by condition. Overall, inequalities in long term health conditions were largest for autism, OR (95%CI), 5.8 (5.0 - 6.6), dementia, 3.1 (2.5 - 3.9), learning disabilities, 2.8 (2.4 - 3.2) and mental health, 2.0 (1.9 - 2.2), with variation by age. In healthcare experience, disparities are much greater for interpersonal communication (15 percentage points lower in adjusted analysis), than access. Additionally, trans and non-binary adults report much higher preference for continuity 1.7 (1.6-1.8), with no evidence of any differences in being able to see or speak to a preferred GP.

Conclusion

This research adds up to date evidence about population demographics, health and healthcare needs, to support healthcare improvement for trans and non-binary adults.

For peer review only

Keywords: primary health care, healthcare quality improvement, long-term conditions, patient experience, trans and non-binary

Strengths and limitations of this study

- Information about the demographics, health, and healthcare needs of people who are trans and non-binary is still emerging; what evidence there is suggests disparities are high
- This study provides nationally representative population-based evidence on long-term conditions and healthcare experiences needed to inform healthcare planning and quality improvement for trans and non-binary adults, particularly in primary care
- Although this work was not fully co-produced the protocol was developed in collaboration with a trans, non-binary and queer patient and public involvement panel, who were involved throughout the whole study

For peer review only

Introduction

Primary care supports populations across the life course, responding to health and healthcare needs that change over time, and with age, and developments in clinical practice. Care for people who are trans and non-binary is one area where GPs are providing health services, while information about the demographics, health, and healthcare needs of the population is still emerging.[1-6]

Addressing gaps in health access and outcomes experienced by minority groups including those with protected characteristics – those characteristics for which it is against the law in the UK to discriminate against someone, and which include gender reassignment - is central to the priorities of the NHS long-term plan.[7,8] Primary care is important for these efforts to prevent ill health and address health inequalities. Clinical guidelines for best practice in trans healthcare to date have only focused on transition or HIV, rather than mental health or primary care.[9] In addition, primary care interventions for trans and non-binary patients have focused on specific areas including ensuring equitable screening access,[10] addressing gaps in secondary care services, and on communication. There is much less evidence about healthcare access and patient experience for non-trans-specific services, and little on the epidemiology of long-term health conditions for trans and non-binary adults, despite these being central dimensions of the work of primary care, and making up the majority of all healthcare contacts.

Data collection within the NHS on trans and non-binary adults and their experiences of healthcare is lacking. To address this evidential need, NHS England is beginning to include questions asking about both gender and trans status in its surveys to support quality improvement programmes.[11,12] We used this data from the GP Patient Survey to answer the research question “what are the demographic characteristics, health conditions, and healthcare experiences of trans and non-binary adults in England?”

For peer review only

Methods

The methods for this cross-sectional secondary data analysis of the GP Patient Survey, including details of the survey data collection,[11] survey tools,[13] measures of gender and trans status, outcomes and outcome coding, statistical analysis and patient and public involvement have been described in full in the pre-registered study protocol,[14] and are reproduced directly from this protocol in an edited form below. The protocol specifies three research questions; of these, RQ1 (“what are the demographic characteristics, health conditions, and healthcare experiences of trans and non-binary adults in England?”) is addressed in this work.[14]

Data

In January 2021 the GP Patient Survey was sent by post to 2,408,303 adult patients registered with a GP in England, from 6694 GP practices, followed by an SMS reminder and two further postal mailings to initial non-responders. Patients from GP Practices with historically low response rates are oversampled. Paper and online responses were possible.

Data were shared with the University of Cambridge under a data sharing agreement with NHS England, and were not linked with any other GP practice or individual data.

Survey Measures

<u>Gender</u>

In 2021, for the first time, the GP Patient Survey included revised questions covering sex, gender and gender reassignment in order for NHS England to meet its duties under the Equality Act (2010) to collect data and address health inequalities in relation to both sex and gender reassignment.

The new questions were developed in consultation with NHS England and stakeholders and were tested in interviews with patients, including trans and non-binary patients, during September 2020. In addition, the questionnaire was reviewed by the Plain English Campaign to meet Plain English criteria; a set of principles designed to ensure information is presented clearly.

The two included questions ask:

“Which of the following best describes you?” with response options “Female”, “Male”, “Non-binary”, “Prefer to self-describe”, and “Prefer not to say”; and, and “Is your gender identity the same as the sex you were registered at birth?” with response options “Yes”, “No”, and “Prefer not to say”.

<u>Long-Term Physical and Mental Health Conditions</u>

Respondents were asked “Which, if any, of the following long-term conditions do you have?” with 17 response options “Alzheimer's disease or other cause of dementia”, “Arthritis or ongoing problem with back or joints”, “Autism or autism spectrum condition”, “Blindness or partial sight”, “A breathing

For peer review only

condition such as asthma or COPD", "Cancer (diagnosis or treatment in the last 5 years)", "Deafness or hearing loss", "Diabetes", "A heart condition such as angina or atrial fibrillation", "High blood pressure", "Kidney or liver disease", "A learning disability", "A mental health condition", "A neurological condition such as epilepsy", "A stroke (which affects your day to day life)", "Another long-term condition or disability", and "I do not have any long term conditions"

Patient Experience

In the GP Patient survey questions ask about experiences of primary care across five domains of healthcare quality: experience before making an appointment, access, continuity, communication and overall experiences of care, with Likert scale response options. For analysis these were categorised into binary (positive / negative) indicators for reporting, in line with national reporting.[5] Full question wording for the 18 survey items included in this analysis, and coding of response options are presented in Appendix Table 1.

Socio-demographic characteristics

We included age, ethnicity, sexual orientation coded from survey responses and a small area (postcode) based measure of socio-economic deprivation (the index of multiple deprivation) categorised into five groups based on quintile defining cut points.

Statistical analysis

In preliminary analyses as per protocol[14] we explored variation by region (the nine government office regions from England), and found little variation across the country except for a slightly higher number of trans and non-binary adults in London, and no impact of region in adjusted models, and so for simplicity we did not include full regional details in our final reporting. We also explored variation across all analyses considering trans and non-binary respondents separately. There are differences in the sizes but not directions of differences between these groups, which warrant further investigation. In this report, in an approach acceptable to the patient and public involvement panel with whom this work was developed,[14] for clarity, we consider all trans and non-binary responses, and additionally responses from people who prefer to self-describe their gender, together in a single group.

To explore demographics of the trans and non-binary population, we described the age, gender, ethnicity, deprivation and sexual orientation of trans and non-binary and all other survey respondents using weighted percentages.

Responses are weighted to the age and gender profile of each GP practice from which responses are sampled, based on information from the sampling frame, to account for design, non-response and calibration to the population of eligible patients. Full details are in the study technical guidance.[11] As GP registration in England is almost universal these weighted estimates can be considered to be nationally representative of the population of England.

To explore long-term health conditions we described the unadjusted weighted percentage of people living with each of 15 physical or mental health conditions, and in adjusted analysis estimated the

For peer review only

odds ratio for reporting each condition for trans and non-binary adults compared with all other survey respondents after adjustment for age, deprivation and ethnicity. Because adjusted analyses include age and gender in the models these are not weighted. In preliminary analyses we additionally considered adjustment for GP practice using a random effect (in order to estimate disparities within GP practices, rather than fixed effect estimates for the population); this had no impact on these models in terms of either effect size, or statistical significance, and so we used fixed effect for population-level estimates, accounting for the GP practice based sampling using cluster robust standard errors.

We also explored whether there was evidence for variation in the relationship between being trans and non-binary and each of the 15 long-term health conditions varied by age, ethnicity, deprivation, sexual orientation and region using an integration term in each model. There was evidence of heterogeneity in the association with trans status for 10 out of the 15 conditions in different age-groups and so we estimated adjusted prevalence stratified by age using recycled predictions from these models, adjusting to the "average" deprivation and ethnicity of all survey respondents.

To explore the healthcare experience of the trans and non-binary population, we calculated the unadjusted weighted percentage of people reporting a positive experience for the 18 patient items. In preliminary analyses we again explored both national (fixed) and within practice (random effect) models. Estimates and standard errors were again the same from both approaches, and estimates from fixed effect models are presented here.

All analyses were carried out using Stata 15.3 Statistical Software (2017) StataCorp, College Station, TX

Patient and Public Involvement

The protocol for this work was developed in collaboration with a trans, non-binary and queer patient and public involvement panel. We met an additional two times online and once in person during this project.

For peer review only

Results

In total 850,206 survey responses were received to the 2021 GP Patient Survey (35% response rate, with 36% of responses online). These included 9,515 where neither gender nor trans status were reported (i.e. either "prefer not to say" or missing responses to both questions), who were excluded from all analyses. Of the 840,691 included responses there were 6,333 trans and non-binary respondents in total (0.9%) who selected either non-binary, or a self-definition for their gender, and/or those who affirmed their gender identity was different from their sex registered at birth and were included in descriptive analyses. We included only responses with complete age, ethnicity and deprivation in multivariable analyses; 827,696 participants, of whom 6091 were trans and/or non-binary. Respondents with missing outcome data from the analyses for long-term health conditions and patient experience on an outcome by outcome basis.

Demographic characteristics

Trans and non-binary adults were younger, more likely to be from Asian, Black, Mixed or Other ethnic groups, less likely to be heterosexual, and are more likely to live in more deprived parts of the country (Table 1, Appendix table 2, and for the multivariable analysis sample only in Appendix table 3).

<Insert Table 1 about here>

For peer review only

Table 1. Respondent characteristics (all respondents, n= 840,691)

	All trans and non-binary respondents (n, %)	All other survey respondents (n, %)
**All respondents (n=840,691)**	6333	834358
**Gender (n=835,561)**		
Female	1708 (28.2)	468958 (56.5)
Male	1971 (32.6)	359266 (43.3)
Non-binary	1220 (20.2)	
Prefer to self-describe	1047 (17.3)	
Prefer not to say	103 (1.7)	1288 (0.2)
**Trans status (n=834,746)**		
Gender identity the same as sex registered at birth	957 (15.3)	825209 (99.6)
Gender identity different from sex registered at birth	4642 (74.4)	
Prefer not to say	644 (10.3)	3294 (0.4)
**Age (n=833,526)**		
16-24	628 (10.1)	41162 (5.0)
25-34	838 (13.5)	69764 (8.4)
35-44	1109 (17.9)	100108 (12.1)
45-54	1155 (18.6)	137231 (16.6)
55-64	1080 (17.4)	174512 (21.1)
65-74	827 (13.3)	172155 (20.8)
75-84	573 (9.2)	132384 (16.0)
**Ethnicity (n=834,261)**		
White	3343 (54.0)	702888 (84.9)
Asian	176 (2.8)	10780 (1.3)
Black	1467 (23.7)	70305 (8.5)
Mixed	482 (7.8)	27612 (3.3)
Other	727 (11.7)	16481 (2.0)
**Sexual orientation (n=820,113)**		
Heterosexual	2682 (46.1)	754746 (92.7)
Lesbian / gay	310 (5.3)	11452 (1.4)
Bisexual	462 (7.9)	8186 (1.0)
Other	752 (12.9)	6235 (0.8)
Prefer not to say	1614 (27.7)	33674 (4.1)
**Deprivation (n=840,691)**		
Most deprived	2211 (34.9)	162804 (19.5)
2	1650 (26.1)	167119 (20.0)
3	1117 (17.6)	172925 (20.7)
4	791 (12.5)	169938 (20.4)
Least deprived	561 (8.9)	161387 (19.3)

For peer review only - http://bmjopen.bmj.com/site/about/guidelines.xhtml

Long-term conditions

The weighted, unadjusted, percentages of each long-term condition among trans and non-binary respondents and all other survey respondents are presented in Table 2, with 39.3% of trans and non-binary respondents and 38.3% of all other respondents reporting no long-term health conditions. However, after adjustment for age, ethnicity and deprivation, overall, for 10 out of the 15 long-term conditions, trans and non-binary adults reported higher prevalence, with inequalities largest for autism, OR (95%CI), 5.8 (5.0 - 6.6), dementia, 3.1 (2.5 - 3.9), learning disabilities, 2.8 (2.4 - 3.2) and mental health, 2.0 (1.9 - 2.2) (Table 2).   Trans and non-binary respondents were 30% less likely to report no long-term conditions after adjustment for age, deprivation and ethnicity, 0.7 (0.7 - 0.8).

<Insert Table 2 about here>

For peer review only

Table 2. Long-term health conditions (735,078 responses, 5110 trans and non-binary)

	All trans and non-binary respondents	All other survey respondents	Adjusted for age, deprivation and ethnicity	p-value
	(n, weighted %)	(n, weighted %)	OR (95%CI)	
Autism or autism spectrum condition	227 (4.4)	4236 (0.6)	5.8 (5.0 - 6.6)	<0.0001
Alzheimer's disease or other cause of dementia	96 (1.9)	5471 (0.7)	3.1 (2.5 - 3.9)	<0.0001
A learning disability	204 (4.0)	7607 (1.0)	2.8 (2.4 - 3.2)	<0.0001
A mental health condition	891 (17.4)	66363 (9.1)	2.0 (1.9 - 2.2)	<0.0001
A stroke (which affects your day to day life)	81 (1.6)	7638 (1.0)	1.8 (1.4 - 2.2)	<0.0001
Blindness or partial sight	118 (2.3)	11955 (1.6)	1.7 (1.4 - 2.0)	<0.0001
Kidney or liver disease	156 (3.1)	17038 (2.3)	1.4 (1.2 - 1.6)	<0.0001
A neurological condition such as epilepsy	116 (2.3)	13855 (1.9)	1.3 (1.1 - 1.6)	0.003
Deafness or hearing loss	287 (5.6)	55229 (7.6)	1.2 (1.1 - 1.4)	0.002
Diabetes	657 (12.9)	72176 (9.9)	1.2 (1.1 - 1.4)	<0.0001
Another long-term condition or disability	645 (12.6)	94872 (13.0)	1.1 (1.0 - 1.2)	0.009
A heart condition such as angina or atrial fibrillation	279 (5.5)	55841 (7.6)	1.0 (0.9 - 1.2)	0.74
A breathing condition such as asthma or COPD	523 (10.2)	89611 (12.3)	1.0 (0.9 - 1.0)	0.28
Arthritis or ongoing problem with back or joints	865 (16.9)	166498 (22.8)	0.9 (0.9 - 1.0)	0.18
High blood pressure	867 (17.0)	158434 (21.7)	0.9 (0.9 - 1.0)	0.055
Cancer (diagnosis or treatment in the last 5 years)	121 (2.4)	31555 (4.3)	0.9 (0.7 - 1.0)	0.092
I do not have any long term conditions	2006 (39.3)	279314 (38.3)	0.7 (0.7 - 0.8)	<0.0001

For peer review only

Adjusted percentages stratified by age are presented in Figure 1 and Appendix table 4 for model estimates, including confidence intervals; in general the differences between trans and non-binary adults (black lines) and all other survey respondents (grey lines) are smaller than the differences between conditions. Trans and non-binary adults are presenting to primary care with similar health profiles to those of all other survey respondents; the age related patterns of long term conditions and variation between conditions is similar across both trans and non-binary respondents and all other survey respondents.

<Insert Figure 1 about here>

Figure 1. Long term condition prevalence, stratified by age. Black line - all trans and/or non-binary respondents; Grey - all other survey respondents

However, despite these over-arching similarities in prevalence, on inspection, there are four different groups of age related patterns across conditions. First, both autism and autistic spectrum conditions, and mental health problems, have higher prevalence at younger ages, and prevalence among trans and non-binary respondents are particularly high at younger ages with absolute differences reducing with age. Second, for dementia, learning disability, blindness, and neurological conditions including epilepsy, adjusted prevalence in trans and non-binary adults follows the same pattern as all other survey respondents, but prevalence shifted upwards with higher prevalence at each age, although absolute differences are small. Third, for stroke, diabetes, and kidney or liver problems the disparities increase with age, with trans and non-binary adults having increasingly higher prevalence compared with all other survey respondents at older age. Finally, for arthritis, hypertension, cancer, breathing problems, heart conditions and deafness, adjusted prevalence in trans and non-binary adults at older ages is lower than prevalence for all other survey respondents (Figure 1, Appendix table 4 for model estimates and Appendix figures 1-3 for versions of Figure 1 with rescaled axes for clarity).

Patient experience

After adjusting for age, ethnicity, deprivation and practice we found that trans and non-binary adults reported slightly poorer overall primary care experiences than all other survey respondents (Table 3) 0.8 (0.8 - 0.9); and across access and overall experience items reported higher numbers of both very good and very poor experiences (Table 4), however there were stronger differences across different domains of patient experience.

<Insert Tables 3 and 4 about here>

For peer review only

Table 3. Patient experience, coding for public reporting

Experience items	Number of responses	All trans and non-binary respondents (n, weighted % positive response)	All other survey respondents (n, weighted % positive response)	OR (95% CI) adjusted for age, deprivation, ethnicity	p-value
**Overall experience**					
Overall (positive) experience	816,702	80.3	85.7	0.8 (0.8 - 0.9)	<0.0001
Overall (positive) experience of making an appointment	752,868	71.4	73.9	1.0 (1.0 - 1.1)	0.84
**Before trying to make an appointment**					
Tried self-management	*742,768*	*30.4*	*31.7*	*0.9 (0.8 - 0.9)*	*<0.0001*
Asked friends or family	*742,768*	*8.4*	*7.7*	*1.0 (0.9 - 1.1)*	*0.53*
Tried online, telephone or other NHS services	*742,768*	*25.0*	*16.8*	*1.3 (1.3 - 1.4)*	*<0.0001*
Tried online or other non-NHS services	*742,768*	*51.8*	*58.0*	*0.9 (0.8 - 0.9)*	*<0.0001*
**Access**					
Found GP practice website easy to use	389,957	70.9	77.1	0.8 (0.8 - 0.9)	<0.0001
Tried to make an appointment in the last 6 months	*777,102*	*66.5*	*64.8*	*1.0 (1.0 - 1.1)*	*0.69*
Getting through on the phone	789,116	69.7	72.5	1.0 (0.9 - 1.1)	0.82
Helpful receptionists	795,619	86.9	90.9	0.9 (0.8 - 0.9)	0.0001
Offered choices when booking appointment	570,565	74.9	71.4	1.2 (1.2 - 1.3)	<0.0001
Satisfied with appointment times available	715,175	73.0	71.0	1.2 (1.1 - 1.3)	<0.0001
Offered an acceptable appointment	749,975	87.4	90.7	0.8 (0.7 - 0.9)	<0.0001
Satisfied with appointment offered	695,269	79.8	83.5	1.0 (0.9 - 1.0)	0.18
Remote appointment (telephone or online)	648,288	53.9	49.2	1.1 (1.1 - 1.2)	<0.0001
**Continuity**					
Have a preferred GP	*776,310*	*64.2*	*50.2*	*1.7 (1.6 - 1.8)*	*<0.0001*
Able to see preferred GP	216,333	59.5	58.8	1.1 (1.0 - 1.2)	0.12
**Communication**					
Involved in decisions about care and treatment	668,530	85.0	93.7	0.5 (0.5 - 0.6)	<0.0001
Had mental health needs in last appointment	*604,671*	*76.2*	*55.5*	*2.1 (1.9 - 2.2)*	*<0.0001*
Mental health needs recognised and understood	336,574	77.6	87.3	0.7 (0.6 - 0.7)	<0.0001
Confidence and trust	744,575	88.7	96.3	0.4 (0.4 - 0.4)	<0.0001
Needs were met	745,767	88.1	95.2	0.5 (0.5 - 0.5)	<0.0001

Experience items marked in *italics* refer to preferences or choices, rather than evaluation of care quality. These questions as well as evaluative items are presented side by side here for clarity across the patient journey

For peer review only

Table 4. Patient experience - very positive and very negative responses

	Adjusted % endorsing the most positive response option		Adjusted % endorsing the most negative response option	
	**Trans and non-binary respondents**	**All other survey respondents**	**Trans and non-binary respondents**	**All other survey respondents**
**Overall experience**				
Overall experience	54.4 (53.1 - 55.6)	53.3 (53.2 - 53.5)	3.3 (2.9 - 3.7)	1.9 (1.8 - 1.9)
Overall experience of making an appointment	41.2 (39.8 - 42.5)	36.3 (36.2 - 36.4)	5.8 (5.2 - 6.4)	4.6 (4.5 - 4.6)
**Access**				
Found GP practice website easy to use	31.9 (30.3 - 33.5)	26.7 (26.6 - 26.8)	9.1 (8.2 - 10.1)	6.8 (6.7 - 6.9)
Getting through on the phone	33.7 (32.5 - 35.0)	25.3 (25.2 - 25.4)	9.8 (9.1 - 10.5)	9.5 (9.5 - 9.6)
Helpful receptionists	56.0 (54.7 - 57.2)	50.3 (50.2 - 50.4)	3.2 (2.8 - 3.6)	2.4 (2.3 - 2.4)
**Communication**				
Involved in decisions about care and treatment	60.4 (59.0 - 61.8)	64.0 (63.9 - 64.1)	11.5 (10.7 - 12.4)	6.3 (6.2 - 6.3)
Confidence and trust	68.3 (67.1 - 69.6)	74.1 (74.0 - 74.2)	8.7 (8.0 - 9.5)	3.7 (3.7 - 3.8)
Needs were met	63.7 (62.3 - 65.0)	67.7 (67.6 - 67.8)	9.1 (8.4 - 9.8)	4.9 (4.8 - 4.9)

For peer review only

There was no evidence of a difference in overall experience of making an appointment, 1.0 (1.0-1.1), with only small differences in experiences across different domains of access (OR range 0.8 to 1.2 across items), however for continuity, trans and non-binary adults were much more likely to report that they had a preference for a particular GP 1.7 (1.6 - 1.8), with no difference compared with all other survey respondents in whether they were able to see this preferred GP or not 1.1 (1.0 - 1.2). Disparities in experiences of interpersonal communication were greatest. After adjustment, trans and non-binary respondents were about half as likely to report a positive experience than all other survey respondents. Trans and non-binary respondents were more likely to report having mental health needs during their appointment 2.1 (1.9 - 2.2), however, among people with mental health needs, trans and non-binary people were less likely to report that these needs had been met (Table 3).

For peer review only

Discussion

Trans and non-binary adults in England responding to the GP Patient Survey reported poorer health and poorer experiences in primary care than all other survey respondents. This research adds up to date evidence in an area where our understanding of the population demographics and healthcare need is still emerging, from a large, nationally representative, sample. This analysis highlights that autism and autistic spectrum conditions, dementia, learning disability and mental health problems are the conditions where disparities are greatest, with variation by age. In healthcare experience, disparities are much greater in interpersonal communication than access. Additionally, trans and non-binary adults report much higher preference for continuity of care than all other survey respondents, with no evidence of any differences in being able to see or speak to a preferred GP. For measures of overall primary care experience, and access, both more very positive and more very negative experiences were reported.

These results, considering both health outcomes and patient experience in primary care, are particularly important in the context of evidence that healthcare outcomes among trans and gender diverse individuals are better when accessing a primary care provider who is knowledgeable about trans health.[15] They also provide insight for understanding the demographics of the trans population in primary care, and the health needs of this group.

Our finding that trans and non-binary adults are over-represented among adults in autism and autistic spectrum conditions, dementia, learning disability and mental health problems in primary care is consistent with previous research which found higher rates of autism, other neurodevelopmental and psychiatric diagnoses in trans and gender-diverse study participants.[16] Our findings of poorer healthcare experiences from trans and non-binary adults also reflect findings from earlier work.[2] Our analyses build on and develop the evidence base from these previous studies, with population-based nationally representative estimates, and provide additional insight about the size of these disparities for most long-term health conditions.

This work provides novel insight into how disparities in long-term health conditions among trans and non-binary individuals vary with age. The four different age-related patterns for long-term conditions point to heterogeneous and potentially intersecting factors driving some of the differences seen. It is interesting to note that this variation in age-related patterns between long-term health conditions is not seen when considering inequalities in long-term health conditions by socio-economic deprivation, for which inequalities consistently increase with age,[17] nor by sexual orientation, where disparities across almost all long-term health conditions are greatest at younger ages and decrease at older ages.[18] For trans and non-binary adults the drivers of differences are likely to be more condition-specific. These patterns are likely, among other possible mechanisms, to be the result of complex interactions between minority stress, behavioural risk factors, and biological effects of exogenous hormones, as well as sexual orientation, socioeconomic status, and healthcare access. In addition,

For peer review only

trans and non-binary people are not a single homogenous group and the patterns observed in long term health conditions may be in part driven by differences across subgroups.

From an epidemiological perspective, it is likely that age, period and cohort effects may all be important in understanding these relationships. For example, the impact of legislation including the Local Government Act (Section 28) in 1988,[19] Gender Recognition Act in 2004,[20] and the Equalities Act in 2010[21] are likely to have had an impact for trans adults during these years (period effects). Mental health, may have been affected by external factors such as transphobic media focus, in more recent years. Long-term gender-affirming therapies may have specific health impacts over time (age effects), and we speculate but cannot confirm that this may be part of the explanation for differences in prevalence of some long-term conditions which are hormonally mediated. Higher mortality among trans and non-binary adults may potentially also have an impact on long-term condition prevalence at older ages, but this cannot be evaluated in this cross-sectional analysis.

We note that trans and non-binary survey respondents were much younger, on average than all other survey respondents, which may suggest that people are more comfortable coming out as trans and at younger ages than previously.[22] The experiences of people who are trans and in their late teens / early 20s in 2021 may be very different from a similarly aged cohort in the past. The much higher prevalence of autism and autistic spectrum conditions and mental health problems among young trans adults compared with older trans adults may reflect these cohort effects as well. Longitudinal population-based data collections are needed to understand causal relationships and properly disentangle the impacts of coming out at different ages on our understanding of these health impacts.[23]

Although the patterns of age-specific long-term health condition prevalence identified provide some insight into disparities experienced by trans and non-binary adults, it is also important to highlight that for most conditions differences in patterns of prevalence between trans and non-binary and all other survey respondents are smaller than differences in prevalence between conditions. This finding highlights that for long-term condition management in primary care the epidemiology of conditions among trans and non-binary adults within a practice is likely to be similar to all other patients within the practice. Standard best practice for the management of long-term conditions, unrelated to trans status, will likely form a large part of the work of GPs with trans and non-binary patients in their practice. Previous work has highlighted that guidelines beyond transition or HIV care still need development.[9] Research areas that underpin long-term condition management, such as ensuring risk prediction models for long-term conditions can be correctly implemented for trans and non-binary patients will remain a priority.[24]

The areas where there are substantial differences in prevalence, however, are for autism and autistic spectrum conditions, and mental health problems, which are both very high among young trans and non-binary adults. In 2016 the Royal College of General Practitioners produced a position statement and best practice guidance, making commitments to enabling access for patients on the autistic spectrum to primary care, and recognising the additional needs of those with autism.[25] Similar

For peer review only

guidance for mental health problems was published in 2017.[26] These guidelines are approaches which, again although not trans-specific, provide practical strategies which may be worth considering for addressing disparities in the poorer healthcare experiences of trans and non-binary patients in primary care.

Our findings describing the patient experience of trans and non-binary patients in primary care provide further insight into where quality improvement work could be directed. The relatively small disparities in access compared with the much larger inequalities in communication experienced by trans and non-binary adults are one such example. Resources are beginning to be developed to support best practice for primary care for communication with trans and non-binary adults in primary care. The 'healthtalk' resource includes interviews with 50 trans and gender diverse young people highlighting individual reflections on how experiences of health care could be improved.[27] Collections of individual experiences provide important insight where evidence-based best practice guidelines are still developing.

The much higher number of trans and non-binary patients who have a preference for continuity is also worth highlighting. Some GPs are regarded by the trans community as providing good trans healthcare and therefore more trans people may register with these GPs[28] whilst surgeries where care is poorer may lose their trans patients. Some trans and non-binary people report having excellent GPs with high levels of training and understanding of trans patients while many do not; this may explain our finding of both very positive and very negative experiences. Our findings that trans and non-binary adults in England are more likely to be living with long-term health conditions, again highlights the importance of continuity.[29] It also highlights that all GPs, regardless of location or special interest are likely to encounter trans and non-binary patients and will be supporting particular needs relating to long term health conditions. Where evidence for this work is lacking further dedicated studies are warranted, with sufficient funding and co-production with the trans and non-binary community.

It is important to identify that there are limitations to the findings from this research; several of these have been discussed in the protocol.[14] From a survey methods perspective, non-differential misclassification of trans and non-binary adults is one concern which has been recognised recently in the development of tools to collect gender and trans status in the NATSAL 4 study.[30,31] Although the number of people incorrectly reporting a trans or non-binary identity will be small, they may form a non-trivial number of trans and non-binary respondents overall. This is one possible explanation for the association seen with dementia, where numbers are low, and inaccuracies (associated with cognitive decline) in inaccurately endorsing a trans and non-binary response may be higher. However the consistency of the findings from this work with previous studies where there were interviewer-led surveys (including, for example, the finding of higher endorsement of both very good and very poor patient experience items)[32] give some reassurance that although this is a concern the impact may be low. A second limitation is the exclusion of respondents who didn't respond, or responded "prefer not to say" to questions about gender and trans-status, it is a concern that these responses may differentially include people who are trans and non-binary. Previous analyses of the GP Patient

For peer review only

Survey focusing on sexual orientation found poorer experiences in these groups.[33] It is also worth highlighting that these findings are limited to trans and non-binary patients who are registered with a GP, and the population who are not registered, or who access primary care only through private GP services such as Babylon,[28] will not be included.

Despite these limitations, the collection of trans status and an inclusive gender question in the GP Patient Survey is an important step towards the generation of evidence for the development of best practice guidance for trans and non-binary patients. Following guidance on delivering good care and communication for those with autism and autistic spectrum conditions, and mental health problems, both of which are prevalent among young trans and non-binary adults, is one way some of the inequalities identified may be addressed. Long-term condition epidemiology, although with some variation between conditions, is broadly similar in both trans and non-binary adults, and all other survey respondents. Good primary care, not just good trans healthcare, is important to address the disparities identified.

For peer review only

Funding

C.L.S. holds a Career Development Fellowship (award number: MH041) funded as part of the Three NIHR Research Schools Mental Health Programme [*]. A.M.M. is funded by the EU/EFPIA Innovative Medicines Initiative Joint Undertaking BigData@Heart grant 116074. This research was supported by core funding from the: British Heart Foundation (RG/13/13/30194; RG/18/13/33946), and NIHR Cambridge Biomedical Research Centre (BRC-1215-20014) [*]. * The views expressed are those of the author(s) and not necessarily those of the NIHR or the Department of Health and Social Care. J.L.'s work for this study is supported by the Wellcome Trust as part of the Wellcome Trust PhD programme for Primary Care Clinicans [grant number 203921/Z/16/Z].

Ethical approval

This is a secondary analysis of anonymised routinely collected data; formal ethical approval is not required

Competing interests

None

Data availability

Person-level data cannot be shared but the tool on the website www.gp-patient.co.uk is excellent for interested researchers to explore the data further

Acknowledgements

Thanks Roary Neat and all other members of the public involvement panel who contributed to this work.

Contributorship Statement

CLS carried out the analysis, wrote the first draft of the paper and is guarantor. CLS, JL, AMM, MR, JS and RD developed the analysis protocol. All authors (CLS, AB, JL, AMM, TO-M, MR, JS and RD) read, critically revised and reviewed the final draft.

ew only

References

1. Ellis SJ, Bailey L, McNeil J. Trans People's Experiences of Mental Health and Gender Identity Services: A UK Study. Journal of Gay & Lesbian Mental Health 2015;19:4-20.

2. Government Equalities Office. National LGBT Survey Research Report. https://www.gov.uk/government/organisations/government-equalities-office. 2018.

3. Stonewall. LGBT in Britain - Trans report https://www.stonewall.org.uk/lgbt-britain-trans-report. 2018.

4. Stonewall. LGBT in Britain - Health https://www.stonewall.org.uk/lgbt-britain-health. 2018.

5. Ipsos MORI, NHS England. GP Patient Survey National Report 2021 Publication https://www.gp-patient.co.uk/surveysandreports. 2021.

6. TransActual. Trans lives survey 2021 https://www.transactual.org.uk/trans-lives-21. 2021.

7. NHS England. The NHS Long Term Plan. https://www.longtermplan.nhs.uk/wp-content/uploads/2019/08/nhs-long-term-plan-version-1.2.pdf. 2019.

8. Ford J, Sowden S, Olivera J, et al. Transforming health systems to reduce health inequalities. Future Healthc J 2021;8:e204-e9.

9. Dahlen S, Connolly D, Arif I, Junejo MH, Bewley S, Meads C. International clinical practice guidelines for gender minority/trans people: systematic review and quality assessment. BMJ Open 2021;11:e048943.

10. Berner AM, Connolly DJ, Pinnell I, et al. Attitudes of transgender men and non-binary people to cervical screening: a cross-sectional mixed-methods study in the UK. Br J Gen Pract 2021;71:e614-e25.

11. Ipsos MORI, NHS. GP Patient Survey 2021 Technical Annex https://www.gp-patient.co.uk/downloads/2021/GPPS_2021_Technical_Annex_PUBLIC.pdf. 2021.

12. Office for National Statistics. Gender identity https://www.ons.gov.uk/census/censustransformationprogramme/questiondevelopment/genderidentity. 2020.

13. Ipsos MORI, NHS. GP Patient Survey - Questionnaire https://www.gp-patient.co.uk/downloads/2021/qandletter/GPPS_2021_Questionnaire_PUBLIC.pdf. 2021.

14. Saunders CL, Lund J, Mason AM, Roberts M, Smith J, Duschinsky R. The Health and Healthcare Outcomes of Trans and/or Non-Binary Adults in England: Protocol for an Analysis of Responses to the 2021 GP Patient Survey. Sexes 2022;3:325-35.

15. Kattari SK, Call J, Holloway BT, Kattari L, Seelman KL. Exploring the Experiences of Transgender and Gender Diverse Adults in Accessing a Trans Knowledgeable Primary Care Physician. Int J Environ Res Public Health 2021;18.

16. Warrier V, Greenberg DM, Weir E, et al. Elevated rates of autism, other neurodevelopmental and psychiatric diagnoses, and autistic traits in transgender and gender-diverse individuals. Nat Commun 2020;11:3959.

17. Barnett K, Mercer SW, Norbury M, Watt G, Wyke S, Guthrie B. Epidemiology of multimorbidity and implications for health care, research, and medical education: a cross-sectional study. Lancet 2012;380:37-43.

18. Saunders CL, MacCarthy S, Meads C, et al. Long-term conditions among sexual minority adults in England: Evidence from a cross-sectional analysis of responses to the English General Practice Patient Survey. BJGP Open 2021;DOI: 10.3399/BJGPO.2021.0067.

19. HM Government. Local Government Act https://www.legislation.gov.uk/ukpga/1988/9/contents. 1988.

For peer review only

20. HM Government. Gender Recognition Act https://www.legislation.gov.uk/ukpga/2004/7/contents. 2004.

21. HM Government. Equality Act https://www.legislation.gov.uk/ukpga/2010/15/contents. 2010.

22. Zhang Q, Goodman M, Adams N, et al. Epidemiological considerations in transgender health: A systematic review with focus on higher quality data. Int J Transgend Health 2020;21(2):125-37

23. MacCarthy S, Saunders CL, Elliott MN. Increased Reporting of Sexual Minority Orientation from 2009 to 2017 in England and Implications for Measuring Sexual Minority Health Disparities. LGBT Health 2020;7:393-400.

24. Berner AM, Lund J, Saunders CL. Tackling the complexity of gender bias in primary care. Br J Gen Pract 2021;71:296-7.

25. Royal College of General Practitioners. RCGP Position Statement on Autistic Spectrum Disorders https://www.rcgp.org.uk/policy/rcgp-policy-areas/autistic-spectrum-disorders.aspx. 2016.

26. Royal College of General Practitioners. RCGP Position Statement on Mental Health in Primary Care https://www.rcgp.org.uk/policy/rcgp-policy-areas/mental-health-in-primary-care.aspx. 2017.

27. healthtalk.org. Experiences of trans and gender diverse young people: GP surgery experiences https://healthtalk.org/Experiences-of-trans-and-gender-diverse-young-people/Trans-and-gender-diverse-young-peoples-experience-of-GP-surgeries. 2022.

28. Action for Trans Health. List of trans friendly GPs https://actionfortranshealth.org.uk/resources/for-trans-people/list-of-trans-friendly-gps/ 2021.

29. Paddison CAM, Rosen R. Tackling the crisis in primary care. Bmj 2022;377:o1485.

30. Connolly A. Presentation at the Taiwan-UK SGS Health Research Network July 17th 2022. 2022.

31. NatCen. Question testing for the National Survey of Sexual Attitudes and Lifestyles 4. Report on findings from cognitive interviews https://www.natsal.ac.uk/sites/default/files/2020-11/Natsal-4-Cognitive-Testing-Report-2019_Publication-1.pdf. 2019.

32. Yarrow E. Gendered bodies: the experiences of gender and sex diverse children and youth in health and education institutions, Centre for Gender Studies, University of Cambridge. 2017.

33. Elliott MN, Kanouse DE, Burkhart Q, et al. Sexual minorities in England have poorer health and worse health care experiences: a national survey. J Gen Intern Med 2015;30(1):9-16

For peer review only

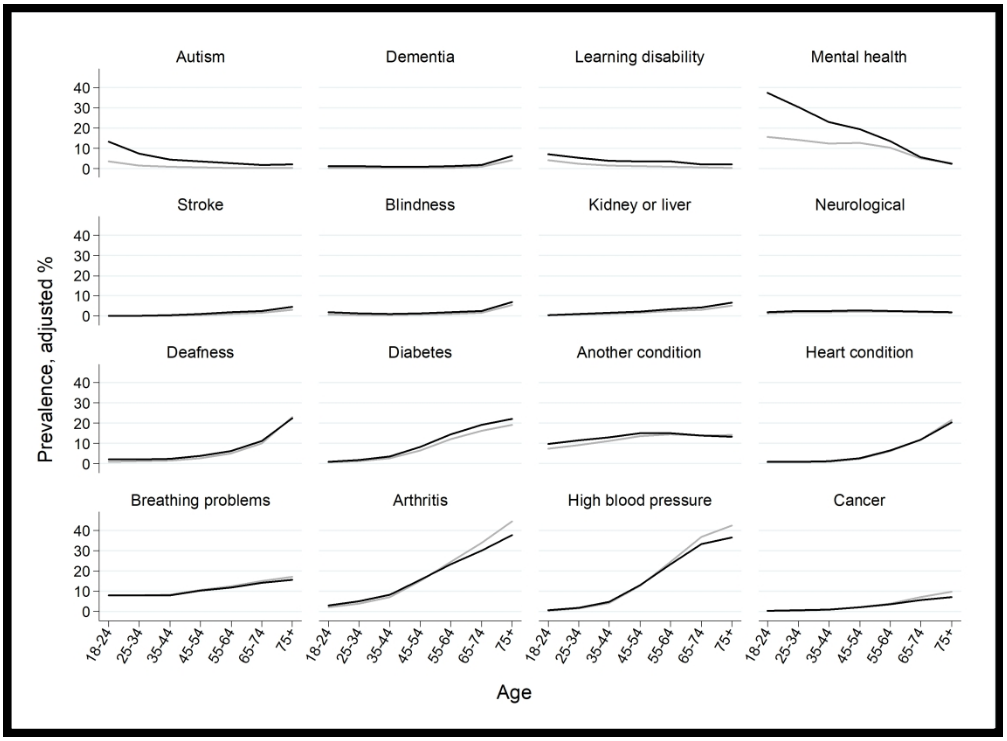

Figure 1. Long term condition prevalence, stratified by age. Black line - all trans and/or non-binary respondents; Grey - all other survey respondents

888x651mm (96 x 96 DPI)

Appendix Table 1. Patient experience, question wording and categorisation for analysis. Reproduced directly from Saunders et al Sexes 2022, 3(3), 325-335; https://doi.org/10.3390/sexes3030025

**Overall experience**	
Overall experience	*Overall, how would you describe your experience of your GP practice?* Very good, Fairly good, compared with Neither good nor poor, Fairly poor, and Very poor
Overall experience of making an appointment	*Overall, how would you describe your experience of making an appointment?* Very good, Fairly good, compared with Neither good nor poor, Fairly poor, and Very poor
**Before trying to make an appointment**	
Tried self-management	*Before you tried to get this appointment, did you do any of the following?* Spoke to a pharmacist, Tried to treat myself/the person I was making this appointment for (for example with medication), compared with all other question respondents *
Asked friends or family	*Before you tried to get this appointment, did you do any of the following?* Asked for advice from a friend or family member, compared with all other question respondents *
Tried online, telephone or other NHS services	*Before you tried to get this appointment, did you do any of the following?* Used an online NHS service (including NHS 111 online), Called an NHS helpline, such as NHS 111, Contacted or used another NHS service, compared with all other question respondents *
Tried online or other non-NHS services	*Before you tried to get this appointment, did you do any of the following?* Used a non-NHS online service, or looked online for information, Tried to get information or advice elsewhere (from a non-NHS service), compared with all other question respondents *
**Access**	
Found GP practice website easy to use	*How easy is it to use your GP practice's website to look for information or access services?* Very easy, Fairly easy, compared with Not very easy, and not at all easy. **
Tried to make an appointment in the last 6 months	*When did you last try to make a general practice appointment, either for yourself or for someone else?* In the past 3 months, Between 3 and 6 months ago, compared with Between 6 and 12 months ago, More than 12 months ago, and I haven't tried to make an appointment since being registered with my current GP practice.
Getting through on the phone	*Generally, how easy is it to get through to someone at your GP practice on the phone?* Very easy, Fairly easy, compared with Not very easy, Not at all easy
Helpful receptionists	*How helpful do you find the receptionists at your GP practice?* Very helpful, Fairly helpful, compared with Not very helpful, and Not at all helpful
Offered a choice when booking appointment	*On this occasion, were you offered any of the following choices of appointment?* Yes, a choice of place (for an appointment in person), Yes, a choice of time or day, Yes, a choice of healthcare professional, Yes a choice of type of appointment (phone call, online, video call, in person), compared with None of these
Satisfied with appointment times available	*How satisfied are you with the general practice appointment times that are available to you?* Very satisfied, Fairly satisfied, compared with Neither satisfied nor dissatisfied, Fairly dissatisfied, and Very dissatisfied
Offered an acceptable appointment	*Were you satisfied with the appointment (or appointments) you were offered?* Yes and I accepted an appointment, No, but I still took an appointment, compared with No, and I did not take an appointment, and I was not offered an appointment
Satisfied with appointment offered	*Were you satisfied with the appointment (or appointments) you were offered?* Yes and I accepted an appointment, compared with No, but I still took an appointment, No, and I did not take an appointment, and I was not offered an appointment
Remote appointment (telephone or online)	*What type of appointment did you get?* I got an appointment… "…to speak to someone on the phone", "…to speak to someone online (for example on a video call)" compared with "…to see someone at my GP practice", "…to see someone at another general practice location", "…for a home visit"
**Continuity**	
Have a preferred GP	*Is there a particular GP you usually prefer to see or speak to?* Yes, for all appointments, Yes for some appointments but not others, compared with No ***
Able to see preferred GP	*How often do you see or speak to your preferred GP when you would like to?* Always or almost always, A lot of the time, compared with Some of the time, and Never or almost never. ****
**Communication**	
Involved in decisions about care and treatment	*During your last general practice appointment, were you involved as much as you wanted to be in decisions about your care and treatment?* Yes, definitely, Yes, to some extent, compared with No, not at all
Had MH needs in last appointment	*During your last general practice appointment, did you feel that the healthcare professional recognised and/or understood any mental health needs that you might have had?* Yes, definitely, Yes to some extent, No, not at all, compared with I did not have any mental health needs
MH needs recognised and understood	*During your last general practice appointment, did you feel that the healthcare professional recognised and/or understood any mental health needs that you might have had?* Yes, definitely, Yes to some extent, compared with No, not at all
Confidence and trust	*During your last general practice appointment, did you have confidence and trust in the healthcare professional you saw or spoke to?* Yes, definitely, Yes, to some extent, compared with No, not at all
Needs were met	*Thinking about the reason for your last general practice appointment, were your needs met?* Yes, definitely, Yes, to some extent, compared with No, not at all

* Only including respondents who have tried to make an appointment with their GP. ** Only including respondents who have tried to use their GP practice’s website. *** Excluding responses from people with only one GP in their practice. **** Only including people with a preference for a particular GP.

For peer review only

Appendix Table 2. Respondent characteristics; row percentages (all responses – comparison with Table 1 in the main paper which presents the same data with column percentages)

	All trans and non-binary respondents (n,%)	All other survey respondents (n,%)
**All respondents (n=840,691)**	6333 (0.9)	834358 (99.1)
**Gender (n=835,561)**		
Female	1708 (0.4)	468958 (99.6)
Male	1971 (0.5)	359266 (99.5)
Non-binary	1220 (100)	
Prefer to self-describe	1047 (100)	
Prefer not to say	103 (7.4)	1288 (92.6)
**Trans status (n=834,746)**		
Gender identity the same as sex registered at birth	957 (0.1)	825209 (99.9)
Gender identity different from sex registered at birth	4642 (100)	
Prefer not to say	644 (16.4)	3294 (83.6)
**Age (n=833,526)**		
16-24	628 (1.5)	41162 (98.5)
25-34	838 (1.2)	69764 (98.8)
35-44	1109 (1.1)	100108 (98.9)
45-54	1155 (0.8)	137231 (99.2)
55-64	1080 (0.6)	174512 (99.4)
65-74	827 (0.5)	172155 (99.5)
75-84	573 (0.4)	132384 (99.6)
**Ethnicity (n=834,261)**		
White	3343 (0.5)	702888 (99.5)
Asian	176 (1.6)	10780 (98.4)
Black	1467 (2.0)	70305 (98.0)
Mixed	482 (1.7)	27612 (98.3)
Other	727 (4.2)	16481 (95.8)
**Sexual orientation (n=820,113)**		
Heterosexual	2682 (0.4)	754746 (99.6)
Lesbian / gay	310 (2.6)	11452 (97.4)
Bisexual	462 (5.3)	8186 (94.7)
Other	752 (10.8)	6235 (89.2)
Prefer not to say	1614 (4.6)	33674 (95.4)
**Deprivation (n=840,691)**		
Most deprived	2211 (1.3)	162804 (98.7)
2	1650 (1.0)	167119 (99.0)
3	1117 (0.6)	172925 (99.4)
4	791 (0.5)	169938 (99.5)
Least deprived	561 (0.3)	161387 (99.7)

For peer review only

Appendix Table 3. Respondent characteristics; (for comparison with Table 1 from main paper; this version includes only the 827,696 responses from the multivariable analysis sample with complete data from all covariates included in multivariable analysis with 6,091 trans and non-binary respondents)

	All trans and non-binary respondents (n, %)	All other survey respondents (n, %)
**All respondents**	6091	827696
**Gender**		
Female	1659 (28.5)	461698 (56.5)
Male	1903 (32.6)	354519 (43.4)
Non-binary	1189 (20.4)	
Prefer to self-describe	981 (16.8)	
Prefer not to say	99 (1.7)	1239 (0.2)
**Trans status**		
Gender identity the same as sex registered at birth	920 (15.3)	813715 (99.6)
Gender identity different from sex registered at birth	4493 (74.7)	
Prefer not to say	599 (10.0)	3073 (0.4)
**Age**		
16-24	622 (10.2)	40967 (5.0)
25-34	826 (13.6)	69415 (8.4)
35-44	1090 (17.9)	99493 (12.1)
45-54	1127 (18.5)	136323 (16.6)
55-64	1061 (17.4)	173233 (21.1)
65-74	809 (13.3)	170965 (20.8)
75-84	556 (9.1)	131209 (16.0)
**Ethnicity**		
White	3300 (54.2)	698007 (85.0)
Asian	170 (2.8)	10656 (1.3)
Black	1442 (23.7)	69465 (8.5)
Mixed	474 (7.8)	27260 (3.3)
Other	705 (11.6)	16217 (2.0)
**Deprivation**		
Most deprived	2127 (34.9)	159640 (19.4)
2	1591 (26.1)	164302 (20.0)
3	1072 (17.6)	170406 (20.7)
4	760 (12.5)	167823 (20.4)
Least deprived	541 (8.9)	159434 (19.4)

For peer review only

Appendix table 4. All age stratified OR and 95%CI (from the model underpinning Figure 1)

	16-24	25-34	35-44	45-54	55-64	65-74	75+
Autism or autism spectrum condition	4.4 (3.4 - 5.6)	5.5 (4.1 - 7.3)	5.4 (3.7 - 7.8)	7.1 (4.9 - 10.3)	10.5 (7.0 - 15.7)	10.5 (5.6 - 20.0)	8.0 (3.3 - 19.6)
Alzheimer's disease or other cause of dementia	2.7 (0.4 - 20.2)	15.2 (8.1 - 28.6)	12.7 (7.5 - 21.4)	6.6 (3.6 - 11.8)	4.7 (2.7 - 8.1)	2.9 (1.7 - 4.8)	1.3 (0.8 - 1.9)
A learning disability	1.9 (1.4 - 2.7)	2.2 (1.6 - 3.0)	2.5 (1.7 - 3.6)	3.7 (2.6 - 5.1)	4.0 (2.8 - 5.8)	7.1 (4.5 - 11.4)	4.6 (2.0 - 10.3)
A mental health condition	4.4 (3.7 - 5.3)	3.0 (2.6 - 3.6)	1.2 (1.0 - 1.5)	1.4 (1.2 - 1.7)	1.5 (1.2 - 1.8)	1.8 (1.4 - 2.4)	1.8 (1.1 - 2.9)
A stroke (which affects your day to day life)	1.6 (1.0 - 2.4)	3.4 (1.1 - 10.7)	3.2 (1.5 - 6.8)	2.2 (1.3 - 3.8)	1.2 (0.7 - 2.1)	2.0 (1.3 - 3.1)	1.6 (1.0 - 2.4)
Blindness or partial sight	2.8 (1.5 - 5.3)	2.5 (1.3 - 4.8)	1.8 (1.0 - 3.4)	2.2 (1.4 - 3.5)	1.6 (1.0 - 2.5)	1.7 (1.1 - 2.6)	1.1 (0.7 - 1.6)
Kidney or liver disease	2.0 (0.7 - 5.4)	0.7 (0.3 - 1.9)	2.0 (1.3 - 3.0)	1.4 (1.0 - 2.1)	1.3 (0.9 - 1.9)	1.1 (0.7 - 1.6)	1.5 (1.0 - 2.1)
A neurological condition such as epilepsy	1.3 (0.6 - 2.5)	2.1 (1.4 - 3.2)	0.9 (0.5 - 1.5)	1.5 (1.0 - 2.2)	1.1 (0.7 - 1.7)	1.5 (0.9 - 2.4)	0.9 (0.4 - 2.0)
Deafness or hearing loss	1.1 (0.5 - 2.5)	2.5 (1.5 - 4.0)	1.9 (1.3 - 2.9)	1.4 (1.0 - 2.0)	1.4 (1.0 - 1.8)	1.1 (0.9 - 1.5)	0.9 (0.7 - 1.1)
Diabetes	0.2 (0.0 - 1.7)	1.2 (0.8 - 2.0)	1.5 (1.1 - 1.9)	1.4 (1.1 - 1.7)	1.1 (0.9 - 1.3)	1.3 (1.1 - 1.6)	1.2 (0.9 - 1.4)
Another long-term condition or disability	1.8 (1.4 - 2.3)	1.7 (1.3 - 2.1)	1.0 (0.8 - 1.2)	0.8 (0.6 - 0.9)	1.1 (0.9 - 1.3)	1.1 (0.9 - 1.4)	1.3 (1.0 - 1.7)
A heart condition such as angina or atrial fibrillation	1.6 (0.6 - 3.8)	1.3 (0.6 - 2.8)	1.2 (0.7 - 2.2)	1.0 (0.6 - 1.5)	1.1 (0.9 - 1.4)	1.0 (0.8 - 1.3)	0.9 (0.7 - 1.2)
A breathing condition such as asthma or COPD	1.2 (0.9 - 1.6)	1.0 (0.8 - 1.3)	0.8 (0.6 - 1.1)	1.0 (0.8 - 1.2)	0.9 (0.7 - 1.1)	1.0 (0.8 - 1.2)	1.0 (0.7 - 1.3)
Arthritis or ongoing problem with back or joints	2.5 (1.7 - 3.7)	1.4 (1.1 - 2.0)	1.3 (1.0 - 1.6)	0.8 (0.7 - 1.0)	0.8 (0.7 - 1.0)	0.8 (0.7 - 1.0)	1.0 (0.8 - 1.2)
High blood pressure	1.3 (0.4 - 4.0)	1.4 (0.8 - 2.3)	1.3 (1.0 - 1.7)	1.0 (0.8 - 1.2)	0.9 (0.7 - 1.0)	0.9 (0.8 - 1.0)	0.8 (0.7 - 1.0)
Cancer (diagnosis or treatment in the last 5 years)	2.4 (0.6 - 9.8)	1.1 (0.4 - 3.5)	1.3 (0.7 - 2.5)	0.7 (0.4 - 1.2)	1.1 (0.8 - 1.5)	0.7 (0.5 - 1.0)	0.8 (0.5 - 1.1)
I do not have any long term conditions	0.3 (0.2 - 0.3)	0.5 (0.4 - 0.6)	0.8 (0.7 - 1.0)	0.9 (0.8 - 1.0)	0.9 (0.8 - 1.0)	0.9 (0.8 - 1.1)	1.2 (0.9 - 1.6)

For peer review only

Appendix figure 1. Dementia, learning disability, blindness and neurological conditions. Long-term condition adjusted prevalence in trans and non-binary adults follows the same pattern as all other survey respondents, but prevalence shifted upwards with higher prevalence at each age

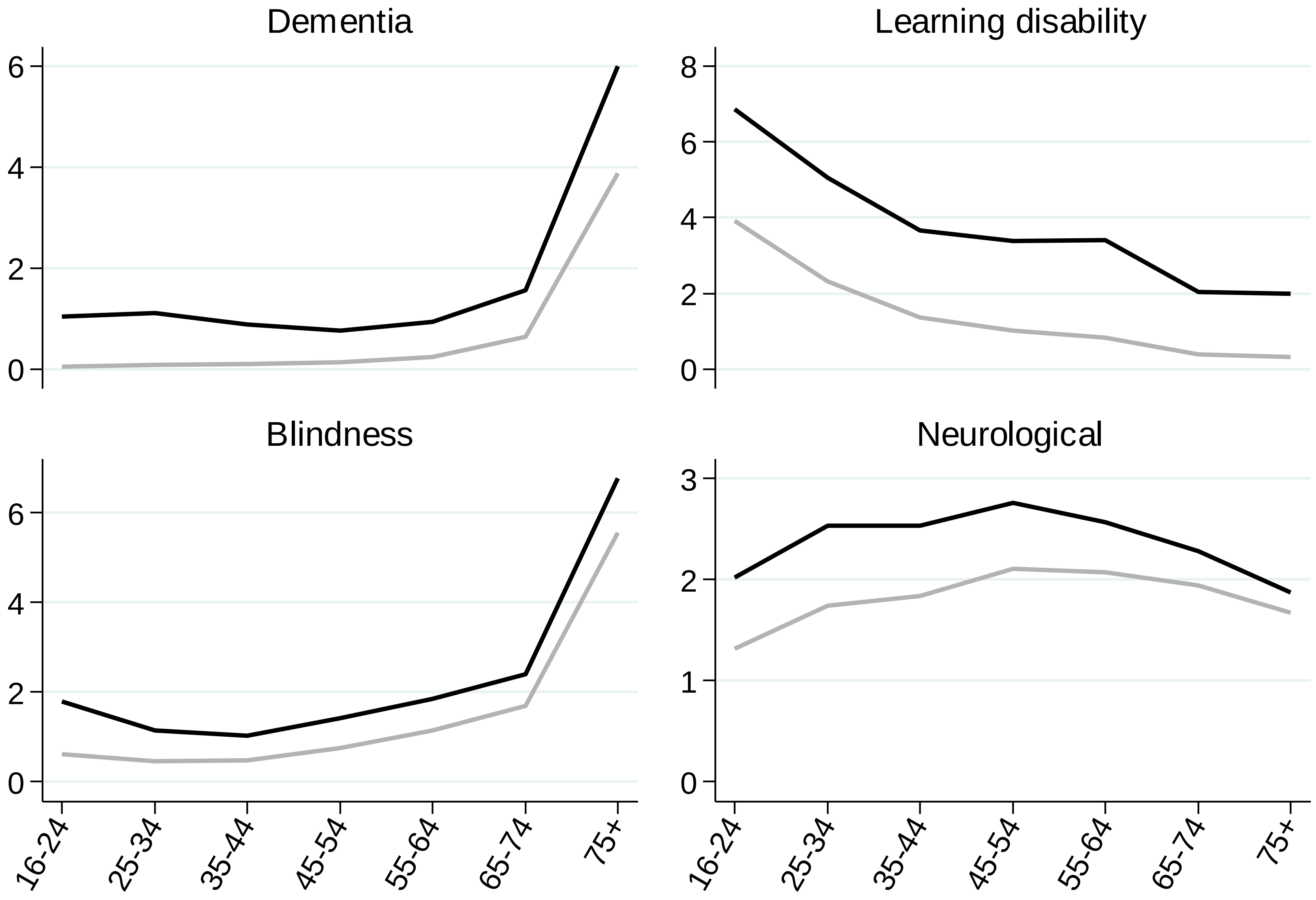

Appendix figure 2. Stroke, kidney or liver problems and diabetes; disparities increase with age, with trans and non-binary adults having increasingly higher prevalence compared with all other survey respondents at older age

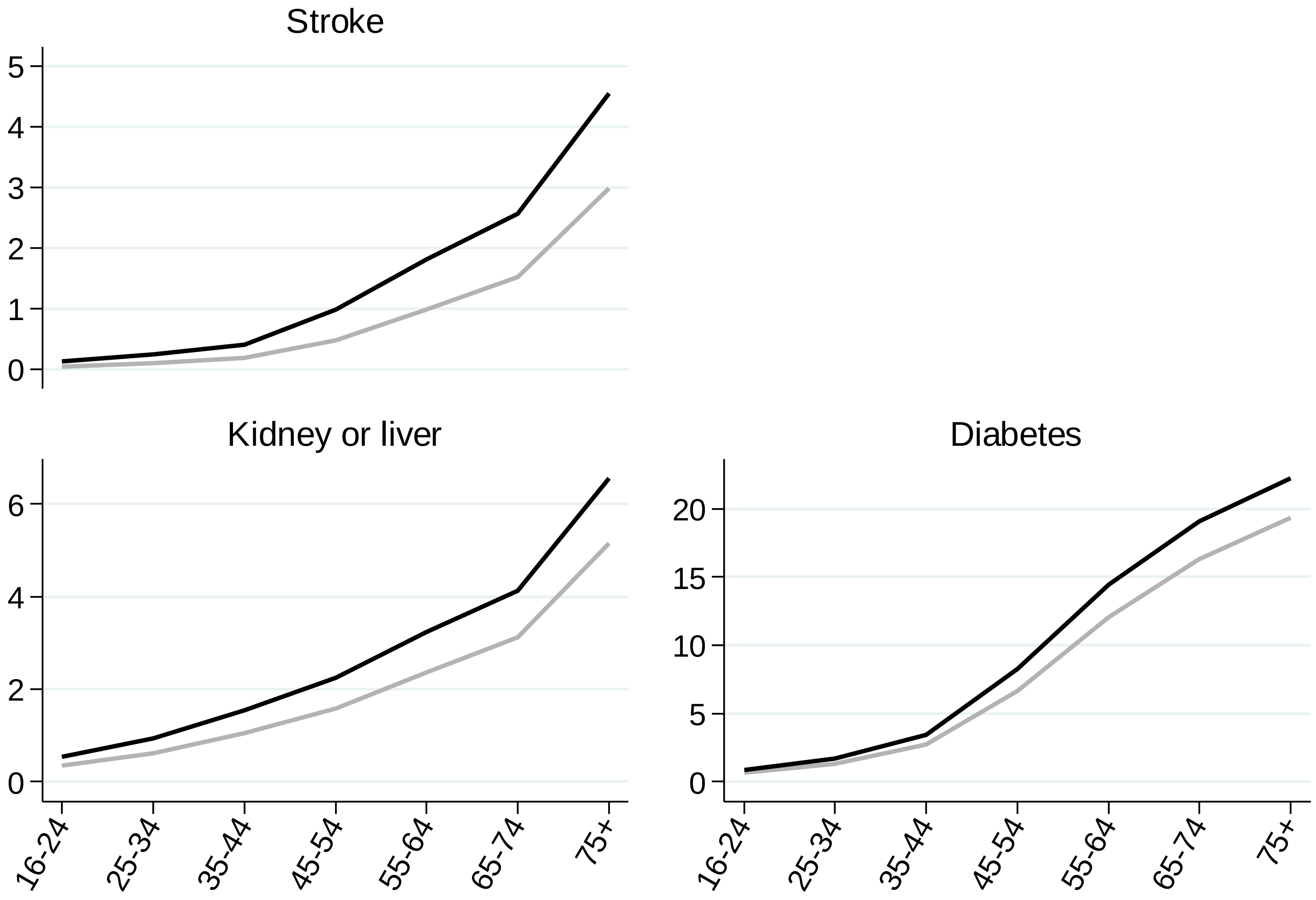

Appendix figure 3. Deafness, heart conditions, breathing problems, high blood pressure and cancer; adjusted prevalence in trans and non-binary adults at older ages is lower than prevalence for all other survey respondents

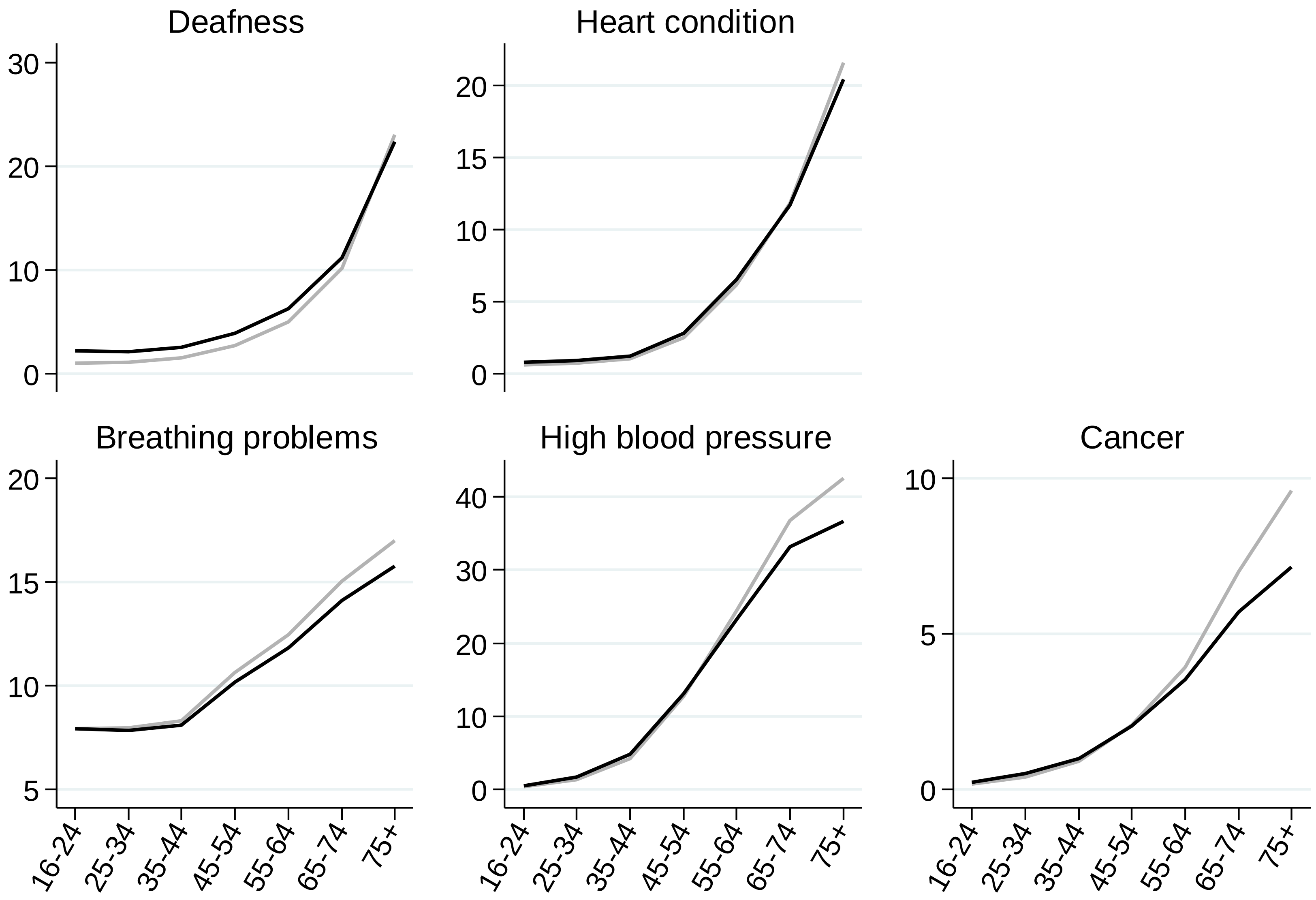

The RECORD statement – checklist of items, extended from the STROBE statement, that should be reported in observational studies using routinely collected health data.

	Item No.	STROBE items	Location in manuscript where items are reported	RECORD items	Location in manuscript where items are reported
**Title and abstract**					
	1	(a) Indicate the study's design with a commonly used term in the title or the abstract (b) Provide in the abstract an informative and balanced summary of what was done and what was found		RECORD 1.1: The type of data used should be specified in the title or abstract. When possible, the name of the databases used should be included.<br><br>RECORD 1.2: If applicable, the geographic region and timeframe within which the study took place should be reported in the title or abstract.<br><br>RECORD 1.3: If linkage between databases was conducted for the study, this should be clearly stated in the title or abstract.	p1, p2
**Introduction**					
Background rationale	2	Explain the scientific background and rationale for the investigation being reported			p4
Objectives	3	State specific objectives, including any prespecified hypotheses			p4
**Methods**					
Study Design	4	Present key elements of study design early in the paper			p5, p6
Setting	5	Describe the setting, locations, and relevant dates, including periods of recruitment, exposure, follow-up, and data collection			p5, plus pre-registered protocol

For peer review only

Participants	6	*(a) Cohort study* - Give the eligibility criteria, and the sources and methods of selection of participants. Describe methods of follow-up<br>*Case-control study* - Give the eligibility criteria, and the sources and methods of case ascertainment and control selection. Give the rationale for the choice of cases and controls<br>*Cross-sectional study* - Give the eligibility criteria, and the sources and methods of selection of participants<br><br>*(b) Cohort study* - For matched studies, give matching criteria and number of exposed and unexposed<br>*Case-control study* - For matched studies, give matching criteria and the number of controls per case		RECORD 6.1: The methods of study population selection (such as codes or algorithms used to identify subjects) should be listed in detail. If this is not possible, an explanation should be provided.<br><br>RECORD 6.2: Any validation studies of the codes or algorithms used to select the population should be referenced. If validation was conducted for this study and not published elsewhere, detailed methods and results should be provided.<br><br>RECORD 6.3: If the study involved linkage of databases, consider use of a flow diagram or other graphical display to demonstrate the data linkage process, including the number of individuals with linked data at each stage.	p5, plus pre-registered protocol
Variables	7	Clearly define all outcomes, exposures, predictors, potential confounders, and effect modifiers. Give diagnostic criteria, if applicable.		RECORD 7.1: A complete list of codes and algorithms used to classify exposures, outcomes, confounders, and effect modifiers should be provided. If these cannot be reported, an explanation should be provided.	n/a
Data sources/ measurement	8	For each variable of interest, give sources of data and details of methods of assessment (measurement).<br>Describe comparability of assessment methods if there is more than one group			p5, plus pre-registered protocol

For peer review only

Bias	9	Describe any efforts to address potential sources of bias			p5, plus details in pre-registered protocol
Study size	10	Explain how the study size was arrived at			p5, plus details in pre-registered protocol
Quantitative variables	11	Explain how quantitative variables were handled in the analyses. If applicable, describe which groupings were chosen, and why			p5, plus details in pre-registered protocol<br><br>(particularly for coding of patient experience items)
Statistical methods	12	(a) Describe all statistical methods, including those used to control for confounding<br>(b) Describe any methods used to examine subgroups and interactions<br>(c) Explain how missing data were addressed<br>(d) *Cohort study* - If applicable, explain how loss to follow-up was addressed<br>*Case-control study* - If applicable, explain how matching of cases and controls was addressed<br>*Cross-sectional study* - If applicable, describe analytical methods taking account of sampling strategy<br>(e) Describe any sensitivity analyses			p6
Data access and cleaning methods		..		RECORD 12.1: Authors should describe the extent to which the investigators had access to the database	P5, plus details in pre-registered protocol

For peer review only

				population used to create the study population.<br><br>RECORD 12.2: Authors should provide information on the data cleaning methods used in the study.	Authors had access to patient-level data under a data sharing agreement with NHS England
Linkage		..		RECORD 12.3: State whether the study included person-level, institutional-level, or other data linkage across two or more databases. The methods of linkage and methods of linkage quality evaluation should be provided.	No
**Results**					
Participants	13	(a) Report the numbers of individuals at each stage of the study (*e.g.*, numbers potentially eligible, examined for eligibility, confirmed eligible, included in the study, completing follow-up, and analysed)<br>(b) Give reasons for non-participation at each stage.<br>(c) Consider use of a flow diagram		RECORD 13.1: Describe in detail the selection of the persons included in the study (*i.e.,* study population selection) including filtering based on data quality, data availability and linkage. The selection of included persons can be described in the text and/or by means of the study flow diagram.	p7, Table 1
Descriptive data	14	(a) Give characteristics of study participants (*e.g.*, demographic, clinical, social) and information on exposures and potential confounders<br>(b) Indicate the number of participants with missing data for each variable of interest<br>(c) *Cohort study* - summarise follow-up time (*e.g.*, average and total amount)			p7, Table 1, Appendix Table 1

For peer review only

Outcome data	15	*Cohort study* - Report numbers of outcome events or summary measures over time<br>*Case-control study* - Report numbers in each exposure category, or summary measures of exposure<br>*Cross-sectional study* - Report numbers of outcome events or summary measures			Full details in Tables 2 and 3
Main results	16	(a) Give unadjusted estimates and, if applicable, confounder-adjusted estimates and their precision (e.g., 95% confidence interval). Make clear which confounders were adjusted for and why they were included<br>(b) Report category boundaries when continuous variables were categorized<br>(c) If relevant, consider translating estimates of relative risk into absolute risk for a meaningful time period			Tables 2-4 and appendix Table 2<br><br>Recycled predictions are presented for clarity
Other analyses	17	Report other analyses done—e.g., analyses of subgroups and interactions, and sensitivity analyses			p7, p8
**Discussion**					
Key results	18	Summarise key results with reference to study objectives			p9
Limitations	19	Discuss limitations of the study, taking into account sources of potential bias or imprecision. Discuss both direction and magnitude of any potential bias		RECORD 19.1: Discuss the implications of using data that were not created or collected to answer the specific research question(s). Include discussion of misclassification bias, unmeasured confounding, missing data, and changing eligibility over	p11

For peer review only

				time, as they pertain to the study being reported.	
Interpretation	20	Give a cautious overall interpretation of results considering objectives, limitations, multiplicity of analyses, results from similar studies, and other relevant evidence			p9, p10
Generalisability	21	Discuss the generalisability (external validity) of the study results			p11
**Other Information**					
Funding	22	Give the source of funding and the role of the funders for the present study and, if applicable, for the original study on which the present article is based			p13
Accessibility of protocol, raw data, and programming code		..		RECORD 22.1: Authors should provide information on how to access any supplemental information such as the study protocol, raw data, or programming code.	p13

*Reference: Benchimol EI, Smeeth L, Guttmann A, Harron K, Moher D, Petersen I, Sørensen HT, von Elm E, Langan SM, the RECORD Working Committee. The REporting of studies Conducted using Observational Routinely-collected health Data (RECORD) Statement. *PLoS Medicine* 2015; in press.

*Checklist is protected under Creative Commons Attribution (CC BY) license.

For peer review only
